# Treatment With the CSF1R Antagonist GW2580, Sensitizes Microglia to Reactive Oxygen Species

**DOI:** 10.3389/fimmu.2021.734349

**Published:** 2021-11-26

**Authors:** Katiria Soto-Diaz, Mario Vailati-Riboni, Allison Y. Louie, Daniel B. McKim, H. Rex Gaskins, Rodney W. Johnson, Andrew J. Steelman

**Affiliations:** ^1^ Neuroscience Program, University of Illinois at Urbana-Champaign, Urbana, IL, United States; ^2^ Department of Animal Sciences, University of Illinois at Urbana-Champaign, Urbana, IL, United States; ^3^ Division of Nutritional Sciences, University of Illinois at Urbana-Champaign, Urbana, IL, United States; ^4^ Carl R. Woese Institute for Genomic Biology, University of Illinois at Urbana-Champaign, Urbana, IL, United States; ^5^ Cancer Center at Illinois, University of Illinois at Urbana-Champaign, Urbana, IL, United States; ^6^ Department of Pathobiology, University of Illinois at Urbana-Champaign, Urbana, IL, United States; ^7^ Department of Biomedical and Translational Sciences, University of Illinois at Urbana-Champaign, Urbana, IL, United States

**Keywords:** microglia, Csf1r, GW2580, reactive oxygen species, *in vivo*, *in vitro*

## Abstract

Microglia activation and proliferation are hallmarks of many neurodegenerative disorders and may contribute to disease pathogenesis. Neurons actively regulate microglia survival and function, in part by secreting the microglia mitogen interleukin (IL)-34. Both IL-34 and colony stimulating factor (CSF)-1 bind colony stimulating factor receptor (CSFR)1 expressed on microglia. Systemic treatment with central nervous system (CNS) penetrant, CSFR1 antagonists, results in microglia death in a dose dependent matter, while others, such as GW2580, suppress activation during disease states without altering viability. However, it is not known how treatment with non-penetrant CSF1R antagonists, such as GW2580, affect the normal physiology of microglia. To determine how GW2580 affects microglia function, C57BL/6J mice were orally gavaged with vehicle or GW2580 (80mg/kg/d) for 8 days. Body weights and burrowing behavior were measured throughout the experiment. The effects of GW2580 on circulating leukocyte populations, brain microglia morphology, and the transcriptome of magnetically isolated adult brain microglia were determined. Body weights, burrowing behavior, and circulating leukocytes were not affected by treatment. Analysis of Iba-1 stained brain microglia indicated that GW2580 treatment altered morphology, but not cell number. Analysis of RNA-sequencing data indicated that genes related to reactive oxygen species (ROS) regulation and survival were suppressed by treatment. Treatment of primary microglia cultures with GW2580 resulted in a dose-dependent reduction in viability only when the cells were concurrently treated with LPS, an inducer of ROS. Pre-treatment with the ROS inhibitor, YCG063, blocked treatment induced reductions in viability. Finally, GW2580 sensitized microglia to hydrogen peroxide induced cell death. Together, these data suggest that partial CSF1R antagonism may render microglia more susceptible to reactive oxygen and nitrogen species.

## Introduction

Microglia comprise 10-15% of total cells in the central nervous system (CNS) and are the recognized resident macrophages of this tissue. As such, microglia function to maintain homeostasis within the healthy brain and act as facilitators of immune responses within the CNS in response to damage or pathogenic challenge. For instance, microglia can clear cellular debris, non-functioning neuronal synapses (1) and dead neurons from the CNS through the process of phagocytosis (2). Upon activation by pathogen associated molecular patterns or by damage associated molecular patterns, microglia release cytokines, chemokines (3, 4) as well as cytotoxic substances including reactive oxygen species (ROS), and nitric oxide (NO) (4–6). While the production of inflammatory mediators by microglia may aid in the clearance of pathogens and/or facilitate repair, aberrant microglia activation is suspected to contribute to a plethora of neurological diseases and disorders including multiple sclerosis (7, 8), Alzheimer’s disease (9), stroke (10), Parkinson’s disease (11), amyotrophic lateral sclerosis (12) and autism spectrum disorder (13, 14). Moreover, pro-inflammatory cytokine production within the CNS is thought to contribute to sickness behavior (15, 16) and impaired cognitive function (15, 17–19).

Intriguingly, microglia viability within the adult brain has recently been shown to be dependent on constitutive colony-stimulating factor 1 receptor (CSF1R) signaling (20) which is needed for their survival, differentiation, proliferation and function (21). CSF1R binds to two ligands: CSF1 and interleukin-34 (IL-34) (22). While CSF1 is detectable in circulation, IL-34 is not circulated in blood (23). In the brain, CSF1 is expressed by astrocytes, oligodendrocytes and microglia, while IL-34 is primarily produced by neurons (24, 25). Ligand binding to CSF1R on microglia leads to tyrosine receptor kinase dimerization and autophosphorylation (26) and triggers activation of ERK and AKT pathways that regulate survival (27). Systemic administration of brain penetrant pharmacological inhibitors of CSF1R can suppress microglia reactivity and/or dramatically reduce viability by more than 90% (28). The use of these small molecules may prove to be clinically efficacious in curtailing the progression of neuroinflammatory and neurodegenerative disease states. Inhibitors of CSF1R include Pexidartinib/PLX-3397, BLZ945, and PLX5622 which eliminate microglia due to their strong affinity with the receptor and their ability to cross the blood brain barrier (28–34). Conversely, the inhibitors ARRY-382, Edicotinib, JTE-952 and GW2580 prevent cell proliferation without killing microglia (35–40). Notably, the consequence of systemic CSF1R antagonism on normal microglia physiology has not yet been fully elucidated. Some of these molecules such GW2580 are intriguing because they are brain penetrant, but do not appear to affect microglial cell survival.

The small molecule GW2580 is an orally available selective inhibitor of the tyrosine kinase activity of CSF1R (39, 40). This molecule has been shown to affect both microglia and monocyte proliferation (40, 41). While GW2580 does not readily affect microglia viability, oral gavage has been used to successfully treat animal models of multiple sclerosis (42, 43), Alzheimer’s disease (44), amyotrophic lateral sclerosis (45) and prion disease (46). Results from these studies suggest that treatment can decrease cell infiltration to the CNS (39, 41) and TNF production (40), however how this drug affects microglia physiology remains unknown.

Herein, we sought to determine the effect of GW2580 on microglia function in healthy mice. We found that treatment did not affect the percentage of circulating immune cell populations. However, our results revealed changes to microglia morphology as well as transcriptomic differences attributable to treatment, such as downregulation of genes related to reactive oxygen species regulation and survival. *In vitro* treatment of primary microglia cultures with GW2580 showed a dose-dependent reduction in viability only when concurrently treated with lipopolysaccharide (LPS). Notably, primary microglial cell cultures treated with either YCG0630, an inhibitor of ROS, or Nec-1, an inhibitor of necroptosis, abrogated the effects of GW2580 and LPS treatment on cell viability. Collectively, our data define the transcriptomic effects of systemic GW2580 treatment on normal microglia. Furthermore, our data suggest that CSF1R antagonism by GW2580 may render microglia more susceptible to reactive oxygen and nitrogen species.

## Materials and Methods

### Animals

All experimental procedures were approved by the Institutional Animal Care and Use Committee at the University of Illinois Urbana–Champaign, under protocol #19068 and were performed in accordance with guidelines of the National Institute of Health. Male and female C57BL/6J (Jackson Laboratories No. 000664) mice aged 8-12 weeks old were used for all experiments. Breeders were group-housed in solid-bottom caging with standard bedding (Teklad 1/8&quot; corncob) under temperature-controlled conditions (23 ± 1°C) with a 12-hour reversed light/dark cycles (10am-10pm). Rodent diet (Teklad No. 8640) and water were available *ad libitum*.

### GW2580 Treatment by Oral Gavage

Oral treatment of rodents with GW2580 has been well characterized (40). Specifically, *in vitro* treatment with GW2580 at 5µM inhibited CSF1 mediated cell proliferation by nearly 100%, and an oral dose of 80mg/kg increased circulating plasma GW2580 concentrations to 5.6µM. Oral treatment was subsequently shown to inhibit disease progression in rodent models of multiple sclerosis [40mg/kg/d (42)], amyotrophic lateral sclerosis [75mg/kg/d; (45)] Alzheimer’s disease [75mg/kg/d; (44)] and to attenuate depression-like behavior in MRL/lpr mice [100mg/kg/d; Chalmers et al. (47)]. As such, in the current study, mice were orally gavaged with either 80 mg/kg of GW2580 (LC Labs, Cat No. G-5903) diluted in 200µl of 0.1% Tween 80, 0.5% hydroxymethyl propyl-cellulose or vehicle alone (44) using 18G needles (Instech, Cat No. FTP-18-30-50) for a total of eight days (40). Animal weight and food disappearance were record daily. Burrowing activity, an indicator of sickness behavior, was measured every 48h (48). On day 8 post treatment, mice were euthanized by CO_2_ asphyxiation, blood was collected *via* cardiac puncture and the mice were perfused with 20-30 mL of sterile phosphate buffered saline (PBS; pH=7.4).

### Flow Cytometry

Following euthanasia blood samples were collected by cardiac puncture with EDTA-coated syringes. Whole blood (100µl) was added to ammonium chloride potassium (500µl) solution for 10 min. at room temperature in order to lyse the red blood cells. The cells were subsequently washed then suspended in ice-cold flow cytometry staining buffer (sterile PBS containing 2% FBS) and counted using an automated cell counter (Nexcelom Bioscience). Next, 1x10^6^ cells were labeled on ice with fluorophore conjugated antibodies to CD4 (Pacific Blue; clone RM4-5; BioLegend, Cat. No. 116008), CD8a (APC-Cy7; Clone 53-6.7; BioLegend, Cat. No. 100714), CD11b (APC; Clone M1/70; BioLegend, Cat. No. 101212), B220 (PE-Cy7; Clone RA3-682; BioLegend, Cat. No. 103224), Ly6G (FITC; Clone 1A8; BioLegend, Cat. No. 127608), Ly6C (PE; Clone HK1.4; BioLegend Cat. No. 128044), and FCblock (CD16/32) for 20 min. Cell were washed, suspended in flow buffer and data were acquired on a LSRII Flow cytometer (BD). Gates were determined using unstained and single-stained samples obtained from the same tissue of origin. Results were analyzed using FlowJo version 10.6.2 flow cytometry software (BD).

Adult brains were collected from mice, minced with a sterile razor blade and an enzymatic digestion for 45 min. in 5ml of StemPro Accutase (Gibco, Cat No. A1110501) at 37°C. Then the brain homogenate was filtered through a 70µm filter using a 3ml syringe plunger, then pelleted by centrifuging at 400x*g* for 5 min. Red cells were removed with a red blood cell lysis buffer (Biolegend, Cat No. 420302) followed by another centrifugation at 400xg for 5 min. Next, cells were suspended in 5ml of PBS solution containing 35% Percoll (VWR, Cat. No. 899428-524), underlay with 3ml of PBS containing 70% Percoll, then centrifuged for 20 min. at 2000x*g* with no brake. The cells at the 35/70% Percoll interface were collected and in some cases these cells were washed with magnetic activated cell sorting (MACS) buffer (PBS with 3% FBS and 10mM EDTA) to enrich for CD11b positive cells following the manufacturer’s instructions (ThermoFisher Scientific, Cat No. 8802-6860-74). Flow cytometry was also used to confirm the purity of microglia subjected to MACS cell enrichment. Here, microglia from non-enriched and enriched populations were stained with antibodies for CD45 (APC; clone 30-F11) (eBioscience, Cat No. 17-0451-82), CD11b (FITC; clone M1/70) (eBioscience, Cat No. 11-0112-82) and viability dye (eFlour 780) (eBioscience, Cat No. 65-0865-14) as markers.

### Immunohistochemistry

Brains were collected and placed in fixation buffer (PBS containing 4% paraformaldehyde, pH 7.4) at 4°C for 24h. The tissue was then cryoprotected by replacing the fixation buffer with PBS containing 30% sucrose (w/v) until the tissue sank. The brain tissue was frozen in optimal cutting temperature compound (O.C.T.) with dry ice, then stored at -80°C. Next, 15µm coronal sections were collected using a cryostat (Leica CM3050 S). Tissue sections were then blocked and permeabilized with PBS containing 5% goat serum and 0.3% Triton X-100, then incubated with an anti-Iba-1 specific antibody derived from rabbit (diluted 1/200; Rabbit; Wako Inc. Richmont, VA, Cat No. 019-19741). After washing with PBS three times for 5 min. the tissues were incubated with an Alexa Fluor 488 conjugated goat anti-rabbit antibody (Invitrogen, Cat No. A11034) diluted in 1/1000 in blocking buffer for 1h at room temperature. After washing, the tissue was coverslipped. Sections were imaged and morphological changes were determined by Imaris surface analysis. Specifically, for the Imaris three-dimensional (3D) modeling and analysis sets of 50 serial images for Iba-1^+^ cells in the cortex at 500 nm steps in the Z direction, 1 µm per pixel in the X, Y-plane, and 4.92 µsec pixel dwell time were acquired with the Zeiss LSM 710 Confocal Microscope and 10x objective with 2.0 zoom, resulting in whole datasets of 425 µm x 425 µm in the X, Y-plane and 25 µm in the Z direction. The images were rendered into 3D with Imaris software (version 9.3.1; Bitplane, Oxford Instruments) and analyzed with the software’s automated Filament Tracer module. Seed point and starting point thresholds were manually adjusted to fit each data set and local contrast threshold was set to three for all data sets. The number of Iba-1^+^ cells from the medial prefrontal cortex, cortex and hippocampus were counted using ImageJ (NIH).

### Isolation of Adult Microglia and RNA Sequencing

Brains were immediately extracted following perfusion with cold PBS and kept on ice in Hanks Balanced Salt Solution (HBSS) buffer (Fisher, Cat No. SH3058801) until further processing. They were minced using as sterile razor blade and enzymatically dissociated for 45 min. in 5ml of StemPro Accutase (Gibco, Cat No. A1110501) at 37°C. The brain homogenate was filtered through a 70µm filter using a 3ml syringe plunger, then pelleted by centrifuging at 400x*g* for 5 min. Red cells were lysed by osmotic shock (red blood cell lysis buffer; Biolegend, Cat No. 420302) then the cells were washed in sterile PBS by centrifugation at 400x*g* for 5 min. Cells were suspended in 5ml of PBS solution containing 35% Percoll (VWR, Cat. No. 899428-524), underlaid with 3ml of PBS containing 70% Percoll, then centrifuged for 20 min. at 2000x*g* with no brake. The cells at the 35/70% Percoll interface were collected and washed with magnetic activated cell sorting (MACS) buffer (PBS with 3% FBS and 10mM EDTA). Microglia were enriched by positive selection using antibodies directed towards CD11b, as previously described. RNA was isolated using the GeneJET™ RNA Purification kit (ThermoFisher Scientific, Cat No. 0732) by following the manufacturer’s instructions. SMART-Seq v4 PLUS Kit (Takara, Cat No. 634888) was used to generate high-quality cDNA libraries from ultra-low amounts of total RNA (10pg–10 ng). Subsequently high-quality Illumina sequencing-ready libraries were prepared and sequencing was performed using with Illumina Hi-seqlane. The fastq read files were generated and demultiplexed with the bcl2fastq v2.20 Conversion Software (Illumina, San Diego, CA, USA). The quality of the resulting fastq files was evaluated with the FastQC software, which generates reports with the quality scores, base composition, k-mer, GC and N contents, sequence duplication levels and overrepresented sequences. On average 47.9 million 150nt paired-end reads per sample were obtained, with a minimum FastQC score of 34. Alignments and counts were performed on the Carl R. Woese Institute for Genomic Biology Biocluster of the University of Illinois High-Performance Biological Computing. Pair-end reads were first filtered using Trimmomatic 0.33 (49) with a minimum quality score of 28 (i.e., base call accuracy of 99.84%) leading and trailing with a minimum length of 30 bp long and subsequently checked using FastQC 0.11.6 (Babraham Institute, Cambridge, UK). No reads were filtered as all had scores greater than 28. Reads were then mapped to the *Mus musculus* reference genome (GRCm38 e!Ensembl, downloaded in September 2019) using default settings of STAR 2.6.0 (50). Uniquely aligned reads were quantified using feature Counts (51) in the Subread package (v1.5.2) based on the Refseq gene annotation.

Further data analysis was conducted using R. 3.5.1 (R Core Team, 2018). Reads uniquely assigned to a gene were used for subsequent analysis. Genes were filtered if 3 samples did not have > 1 count per million mapped reads. A TMM (trimmed mean of M-values) normalization was applied to all samples using edgeR (52). After data were log2-transformed, edgeR was used to conduct differential expression analyses. The applied statistical model included treatment (GW2580 vs Control) as fixed effect.

The genome index was prepared using STAR and the *Mus musculus* DNA FASTA file from e!Ensembl. The reads were aligned to the reference genome to both reads. Reads were counted using a FeatureCount in STAR. Statistical analysis of differentially expressed genes (DEG) was performed using R studio. Gene ontology was determined by DAVID Functional AnnotationBioinformatics Microarray Analysis (v6.8). Data is publically available through Gene Expression Omnibus (GEO) from NCBI at (https://www.ncbi.nlm.nih.gov/geo/query/acc.cgi?acc=GSE185564) and can be accessed using the accession no. GSE185564

### Primary Microglia Cultures

Glial cultures were prepared from the brains of 4-10 C57BL/6 neonatal mice per experiment using the differential attachment method as described previously (3, 53). In brief, P1–2 mouse pups were decapitated with scissors, brains dissected and meninges removed under a dissection microscope (Leica). Brain tissue was disassociated in Accutase at 37°C for 45 min., passed through a 70μm filter, washed with Dulbecco’s Modified Eagle Medium (DMEM), seeded onto poly-d-lysine-coated T75-flasks, and grown to confluence (7–10 days) in a humidified incubator at 37°C and 5% CO_2_. Microglia were isolated by shaking the T-flasks at 37°C for 1h at 177rpm in an orbital shaker (ThermoScientific Max Q 4000).

### Effect of GW2580 Treatment on Microglia Proliferation

Primary microglia were seeded at a density of 1x10^5^ cells per well in a 96-well plate in DMEM supplemented with 10% FBS, 100U pen/strep and glutamax. To determine the effect of CSF1 and GW2580 on cell proliferation, microglia were treated with recombinant mouse CSF1 (0-30ng/ml; R&D Systems; Cat No. 416-ML-10) for 48h with increasing concentrations of GW2580 (0-5μM). After treatment, cells were fixed with 4% paraformaldehyde for 20 min. at room temperature, washed with PBS then blocked and permeabilized in PBS containing 0.3% Triton-X 100 (PBST) and 5% goat serum for 1h at room temperature. Next, the cells were incubated with a rabbit anti-Ki-67 antibody (Dilution 1:200; Cell Signaling; Cat No. 9129S) overnight at 4°C. After washing with PBS, cells were incubated with Alexa Fluor 488 conjugated goat anti-rabbit IgG diluted 1/1000 in PBST for 1h. (ThermoScientific). After washing with PBS, proliferation was determined by counting the number of microglia per well, as well as the number of Ki-67^+^ cells per well using ImageJ (NIH).

### Gene Expression Analysis by qRT-PCR

To validate the effect of GW2580 on the expression of select genes involved in the regulation of ROS, qRT-PCR was used. Here, primary microglia were stimulated with 5µM of GW2580 or media alone for 72h in a humidified incubator at 37°C and 5% CO_2_. RNA was isolated using TRIzol protocol (Ambion by Life Technologies, Cat No., 15596018) and further purified using the GeneJET RNA Purification Kit (Thermo Scientific, Cat No., K0731). cDNA was created using the reverse transcription system (Promega Corporation, Cat No., A3500), and amplified using Sybr Green qPCR master mix (Applied Biosystems, Cat No., A25742), and primers specific for *Ndufs8* (Fwd-CGCAGCACTTCAAGATGTATCG; Rev-TCTTGGCTCAGCCTCAATGG), *Prdx2* (Fwd-CAACCACCGCCAGAATTGC; Rev-AACATTGTGGATGGCTTGGC), *Prdx5* (Fwd-AGGGTACCCAACCCTGTTCT; Rev-GCACAGTAGTACACAGCCGA), *Gpx4* (Fwd-CGCCAAAGTCCTAGGAAACG; Rev-TATCGGGCATGCAGATCGAC), and *Actb* (Fwd-GATTACTGCTCTGGCTCCTAG; Rev-GACTCATCGTACTCCTGCTTG) in a 7300 Real-Time PCR System (Applied Biosystems, ABI) at 95°C for 10min, followed by 40 cycles of 95°C for 15secs and 60°C for 1min. Expression was normalized to *Actb* expression and control samples. Fold change was calculated using the formula 2^-ΔΔCt^.

### Detection of Reactive Oxygen Species (ROS) in Cultured Microglia

Primary microglia (1x10^6^ per condition) were stimulated with 100ng/ml of LPS for 0, 6 or 24h in a 1.5ml tube in complete RPMI media at 37°C and 5% CO_2_. Cells stimulated with media containing 75µM hydrogen peroxide were used as a positive control. After the stimulation, light-protected cells were stained with 5µM of CellROX Green Reagent (ThermoFisher Scientific, Cat No. C10444) for 30 min. at 37°C and 5% CO_2._ The cells were washed twice with 1ml of cold flow buffer (PBS, 2% FBS) then suspended in 100µl of flow buffer. The cells were stained with viability dye (APC Alexa Flour 780, Invitrogen, Cat No. 50-112-9035) for 20 min on ice, washed, then suspended in 500µl of flow buffer and analyzed using flow cytometer. Gates were determined using a complete unstained sample.

### Microglia Susceptibility to ROS

To assess the effect of GW2580 on cell viability, microglia were incubated with or without GW2580 at a concentration of 5µM for 45 min. prior to stimulation with vehicle (medium) or lipopolysaccharide from *Escherichia coli* O111:B4 (100ng/ml) for 48h. TNF production was measured by ELISA (ThermoFisher Scientific, Cat No. 88-7324-88) following the manufacturer’s instructions. For some experiments, microglia were pre-treated with the ROS inhibitor YCG063 (10µM, Sigma Aldrich, Cat No. 557354). Specifically, cells were pre-treated with either YCG063 or YCG063 plus GW2580 for 45 min. prior to LPS stimulation for 48h. To determine if GW2580 treatment of microglia increased sensitivity to ROS, cells were pretreated with GW2580 (5μM) for 48h then challenged with 0-100µM of hydrogen peroxide (H_2_O_2_; Fisher Scientific, Cat No. H325-100) for 4h. Cell death was assessed by measuring supernatant lactate dehydrogenase (LDH) levels as indicated by manufactures instructions (Sigma Aldrich, Cat No. 4744926001) as well as by counting the number of cells per field.

### Treatment of Primary Microglia With Nec-1

Primary microglia (1x10^5^ per condition) were pre-treated with either Nec-1 (30nM) or Nec-1 plus GW2580 for 45 min. prior to LPS (100ng/ml) stimulation for 48h in a 96 well plate. After treatment, cells were fixed with 4% paraformaldehyde for 20 min. at room temperature, washed with PBS then blocked and permeabilized with PBS containing 0.3% Triton-X 100 (PBST) and 5% goat serum for 1h at room temperature. Next, the cells were incubated with a rabbit anti-Iba-1 antibody (diluted 1/200; Rabbit; Wako Inc. Richmond, VA, Cat No. 019-19741) overnight at 4°C. After washing with PBS, cells were incubated with Alexa Fluor 488 conjugated goat anti-rabbit IgG diluted 1/1000 in PBST for 1h. (ThermoScientific). Cell death was assessed by measuring supernatant lactate dehydrogenase (LDH) levels as indicated by manufacturer’s instructions (Sigma Aldrich, Cat No. 4744926001).

### Statistics

Data are expressed as means ± standard error (S.E.). For the RNA-seq experiment differentially expressed genes (DEG) across time points were determined with a combination of fold-change (> 2.0 or < -2.0) and *P*-value (< 0.05) thresholds to balance for reproducibility, sensitivity, and specificity or results (54, 55). Statistical analysis of all other data was performed using GraphPad Prism (version 8.0 or higher) software. Student’s *t*‐tests were used to compare differences between two groups. Analysis of variance (ANOVA) followed by Bonferroni’s *post hoc* test was used to compare differences between more than two groups. Statistical significance was determined by p-value <0.05. Data are expressed as means ± standard error (S.E.).

## Results

### Effect of GW2580 Treatment on Indices of Sickness and Circulating Immune Cell Populations

**Figure 1:**
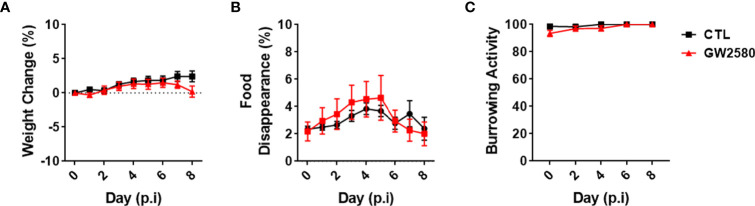
Effect of GW2580 treatment on mouse weight and behavior. **(A–C)** Mice were treated with GW2580 at a dose of 80mg/kg/d by oral gavage for 8 days. The effect of treatment on weight **(A)**, food disappearance **(B)**, and burrowing activity **(C)** is shown. Data are expressed as means ± standard error (S.E.). Each group is composed by n=30 per group from four independent experiments for weight change, n=6 per group from a single experiment for food disappearance, and n=15 per group from three independent experiments for the burrowing activity.

**Figure 2:**
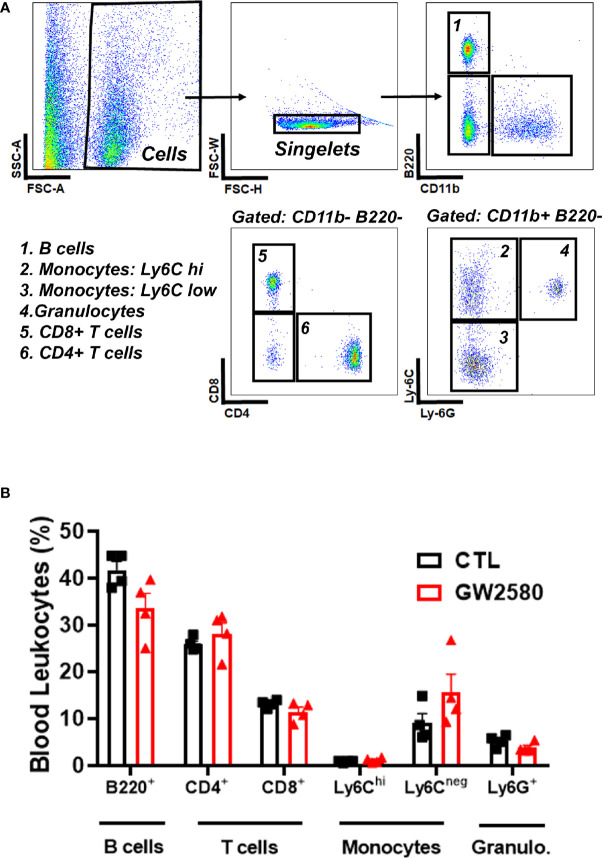
Effect of GW2580 treatment on blood leukocytes. **(A, B)** Mice were treated with vehicle or GW2580 by oral gavage for 8 days and the effect of treatment on circulating leukocytes determined by flow cytometry. **(A)** Representative gating strategy used to determine the percentage of B cell, T cell, monocyte and granulocyte subsets. **(B)** Cell percentages of blood leukocytes from vehicle and treated mice are shown. Data are expressed as means ± standard error (S.E.), n=4 per group.

Treatment with GW2580 neither reduced body weight nor the degree of food disappearance (**Figures 1A, B**). Furthermore, GW2580 did not alter burrowing behavior (**Figure 1C**). No effect of gender was found for weight, food intake or burrowing behavior (**Supplemental Figure 1**). Because macrophages and monocytes also express *Csf1r* (56), we questioned whether treatment affected the percentages of circulating immune cells. Flow cytometry analysis performed on blood samples indicated that treatment did not alter percentages of circulating B cells, T cells, monocytes or granulocytes (**Figure 2**). Together, these data indicate that treatment with GW2580 does not overtly affect behavior or hematopoiesis, nor does it decrease blood leukocyte viability.

### Treatment With GW2580 Caused Morphological and Transcriptomic Alterations to Microglia

**Figure 3:**
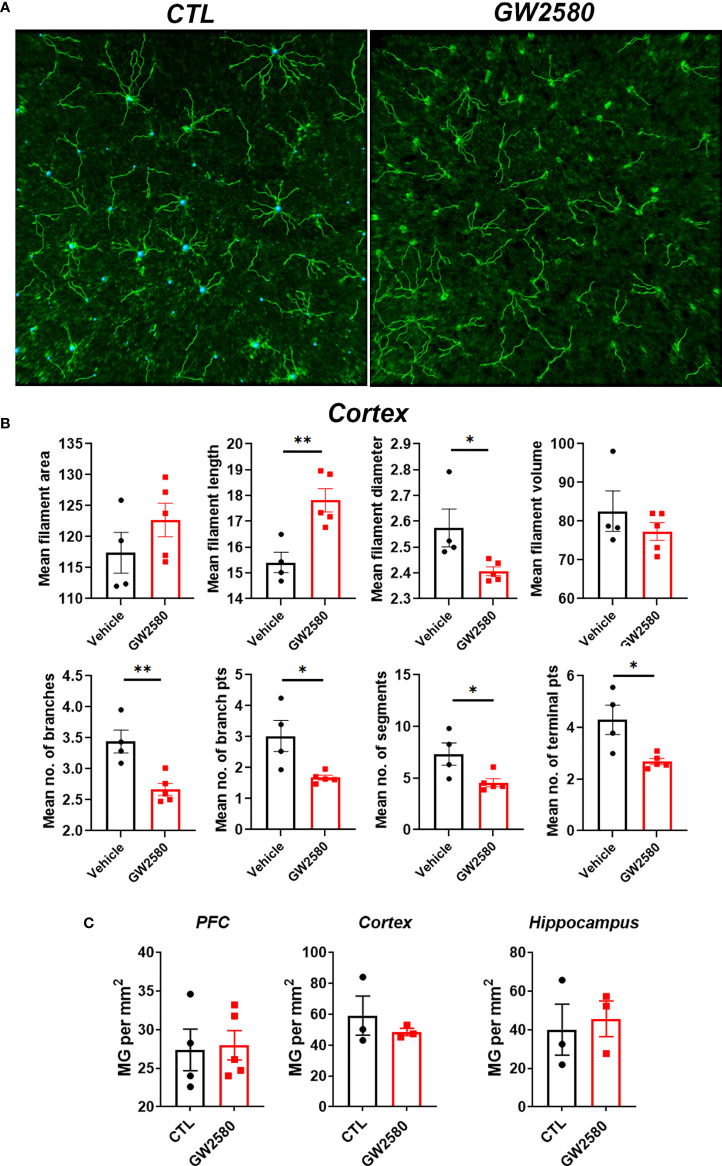
Effect of GW2580 treatment on changes to microglia morphology. **(A–C)** Mice were treated with GW2580 by oral gavage for 8 days and the effect of treatment on microglia morphology and viability examined.** (A)** Representative three dimensional reconstruction of a microglia cell in the cortex of vehicle and GW2580 treated mice from a 20x picture. Scale bar is 50μm. **(B)** Results from Imaris analysis. Each point represents an average of three cells per mouse (n=4-5 animals per group), average of three 20x z-stacks of 25µm and 500nm steps per animal. **(C)** Microglia counts per mm^2^ in the prefrontal cortex, cortex and hippocampus. Each point represents an average of three pictures. Data shown are expressed as means ± standard error (SE). Statistical significance is represented by p-value < 0.05 (*), and p-value < 0.01 (**).

We next questioned whether treatment with GW2580 altered microglia morphology, a commonly used proxy for activation. Imaris™ software was used to non-subjectively determine effects of GW2580 on various morphological characteristics of Iba-1 labeled microglia in 20x images including: mean filament area, filament length, filament diameter, filament volume, mean number of branches, mean number of branch points, mean number of segments and mean number of terminal points (**Figure 3A** and **Supplemental Table 1**). The cortex was chosen as a representative region of morphological changes after GW2580 treatment (57). We found that GW2580 treatment altered morphological characteristics of brain microglia in the cortex. Specifically, the filament length was increased, but filament diameter, number of branches, number of branch points, the number of segments and the mean number of terminal points were decreased as a result of GW2580 treatment (**Figure 3B**). Interestingly, morphological analysis of Iba-1^+^ microglia in 40x images showed similar results, where the number of branch points were decreased in the treated mice, as well as the number of segments (**Supplemental Figure 2**). However, as in previous studies (40), GW2580 treatment for 8 days did not change the number of microglia in medial prefrontal cortex, cortex or cerebellum (**Figure 3C**).

**Figure 4:**
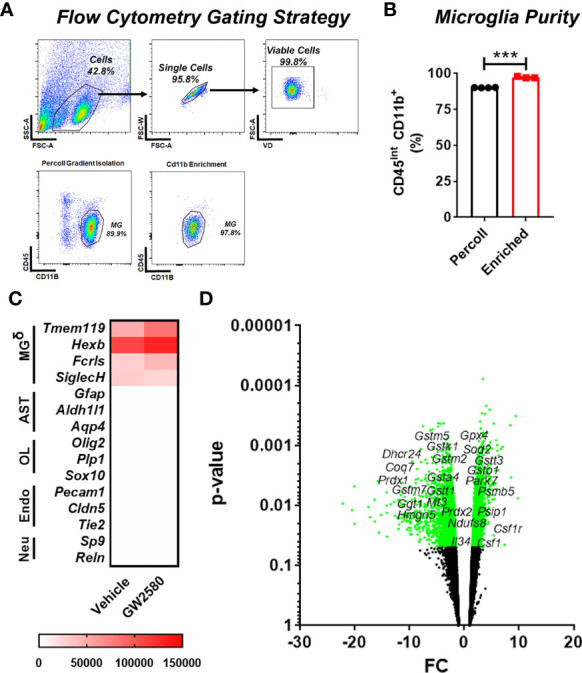
Microglia transcriptional changes induced by GW2580 treatment. **(A–D)** Mice were gavaged with vehicle or GW2580 for 8 days then brains were collected, and microglia were isolated by Percoll gradient centrifugation, enriched by positive selection by magnetic associated cellular sorting (MACS) using antibodies specific for CD11b and changes to the transcriptome assessed by RNA-seq. **(A, B)** Microglia purity was determined before (Percoll) and after (enriched) MACS enrichment by flow cytometry. Representative flow cytometry gates **(A)** and cell purity following Percoll gradient centrifugation and positive selection **(B)**. **(B)** n=3-4 mice per condition. **(C)** Mean expression values of select microglia, astrocyte, oligodendrocyte, endothelial and neuron specific cell lineage genes following transcriptomic analysis of microglia isolated from vehicle (*left*) and treated (*right*) mice (n=5 per condition). Scale bar is gene expression level (RMPK). δ= Genes for microglia were enriched. **(D)** Volcano plot representing all the genes detected with the RNA sequencing experiment, significant genes are represented in green, including *Csf1, Csf1r* and *Tspo*, as well as the ROS related genes. Data are expressed as means ± standard error (S.E.). Statistical significance is represented by p-value < 0.001 (***).

To access the *in vivo* effects of CSF1R inhibition, we performed RNA sequencing on freshly isolated microglia from GW2580 treated mice. Microglia were enriched by MACS using CD11b microbeads. This produced greater than 95% enrichment of cells phenotypically characterized as being CD11b^+^CD45^int^ by flow cytometry (**Figures 4A, B**). The majority of the contaminating cells (5%) were found to be CD11b^+^CD45^hi^ monocytes/granulocytes (**Figures 4A, B**). To validate the purity of microglia after RNA-seq was performed, we examined the expression of genes specific for microglia (*Tmem119*, *Hexb*, *Fcrls*, *Siglech*), astrocytes (*Gfap*, *Aldh1l1*, *Aqp4*), oligodendrocytes (*Olig2*, *Plp1*, *Sox10*), endothelial cells (*Pecam1*, *Cldn5*, *Tie2*) and neurons (*Sp9*, *Reln*) (**Supplemental Table 2**). This analysis revealed that the RNA was enriched for microglia specific genes, confirming that the sequenced cells were microglia (**Figure 4C** and **Supplemental Table 2**). In total, we identified 577 differentially expressed genes (DEGs), of which 144 were upregulated and 433 were downregulated. Since GW2580 is a CSF1R antagonist we hypothesized that genes involved in the CSF1/CSF1R signaling pathway may be affected by treatment. Therefore, we examined the expression values of the CSF1R ligands *Csf1* and *Il34* as well as the expression of *Csf1r* itself. The RNA sequencing analysis showed that *Csf1* was upregulated in the GW2580 treated group. Furthermore, while the mean expression value for *Csf1r* trended towards being higher in the treated group, the effect did not reach statistical significance (p-value of 0.08). Consistent with the fact that *Il34* is predominantly expressed by pericytes and neurons (Tabula Muris Consortium, 2018) but not microglia, we found the expression values for *Il34* were low. There was no difference in the level of *Il34* expression. Analysis of the gene ontology terms indicated that genes encoding proteins involved in glutathione metabolic process, response to oxidative stress, removal of superoxide radicals and response to estradiol were decreased by treatment (**Figure 4D** and **Supplemental Table 3**). Additionally, ontology analysis of the upregulated genes showed that GW2580 affects expression of genes involved in G-protein coupled receptor signaling pathway and sensory perception of smell (**Supplemental Table 4**).

### *In Vitro* Effects of Inhibiting Csfr1 From Primary Microglia After GW2580 Treatment

**Figure 5:**
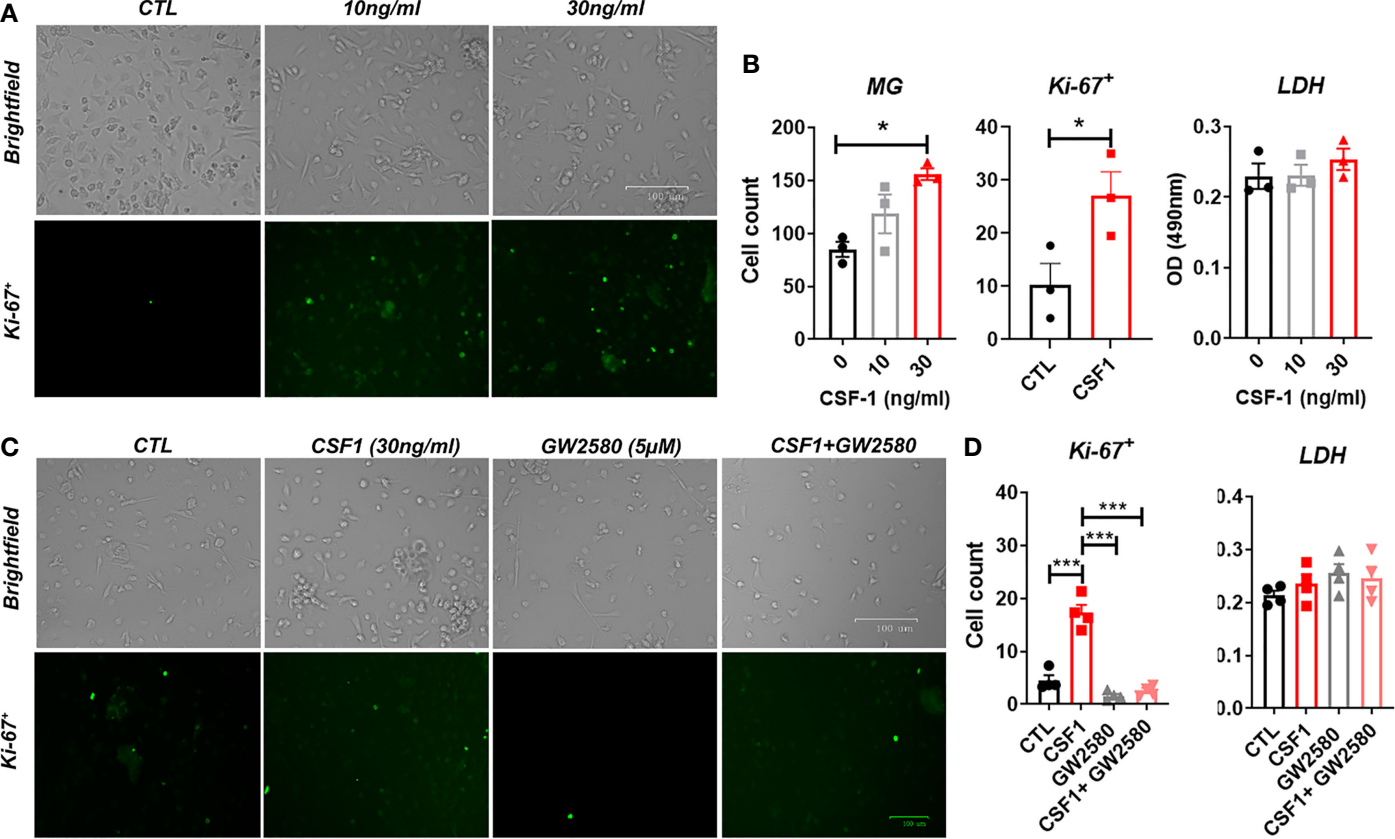
Effect of GW2580 treatment on microglia cell proliferation. **(A, B)** Primary mouse microglia were stimulated with 0, 10 or 30ng/ml of CSF1 and then stained with 30µg/ml of Ki-67. Cell death was assessed by measuring supernatant levels of LDH. Cells were manually count and represented by the graphs. Data are expressed as means ± standard error (S.E.), n=3 independent experiments per group. **(C, D)** Cells were treated with either media, with CSF1 (30ng/ml), GW2580 (5µM), or CSF1 and GW2580, then Ki-67. The number of Ki-67^+^ cells determined by immunohistochemistry were manually counted and represented by the graphs. Data are expressed as means ± standard error (S.E.), n=4 independent cultures per group. Statistical significance is represented by p-value < 0.05 (*), and p-value < 0.001 (***).

We sought to determine if GW2580 treatment affected microglia in culture. Unlike microglial cell lines, such as BV-2 cells, primary mouse microglia monocultures do not readily proliferate in culture. However, as reported by others (58, 59) we found that CSF1 treatment for 48h increased cell number as well as the number of Ki-67^+^ cells per well in a dose-dependent fashion without affecting cell viability (**Figures 5A, B**). These data indicate that primary microglia are responsive to CSF1. To establish the dose at which GW2580 affects microglial function, primary cultures were pretreated with increasing concentration of GW2580 then stimulated with either media or CSF1. As before, CSF1 treatment increased the number of Ki-67^+^ cells. However, concurrent treatment with GW2580 ameliorated this effect, without affecting cell viability (**Figures 5C, D**). These findings indicate that cultured primary microglia proliferate in response to CSF1 and confirm that treatment with GW2580 at a concentration of 5μM is sufficient to inhibit the effects of CSF1 signaling.

To confirm the effects of GW2580 on cultured microglia, we examined the expression levels of select genes that comprised the gene ontology term “response to oxidative stress” (*Ndufs8, Prdx5*), “removal of superoxide radicals” (*Prdx2*), and “glutathione metabolic process” (*Gpx4*), identified by our RNA-seq analysis. Treatment of primary microglia cultures with GW2580 suppressed the expression of *Ndufs8*, *Prdx2*, *Prdx5* and *Gpx4* without affecting viability (**Supplemental Figure 3**).

### Treatment With GW2580 Sensitizes Microglia to ROS Toxicity

**Figure 6:**
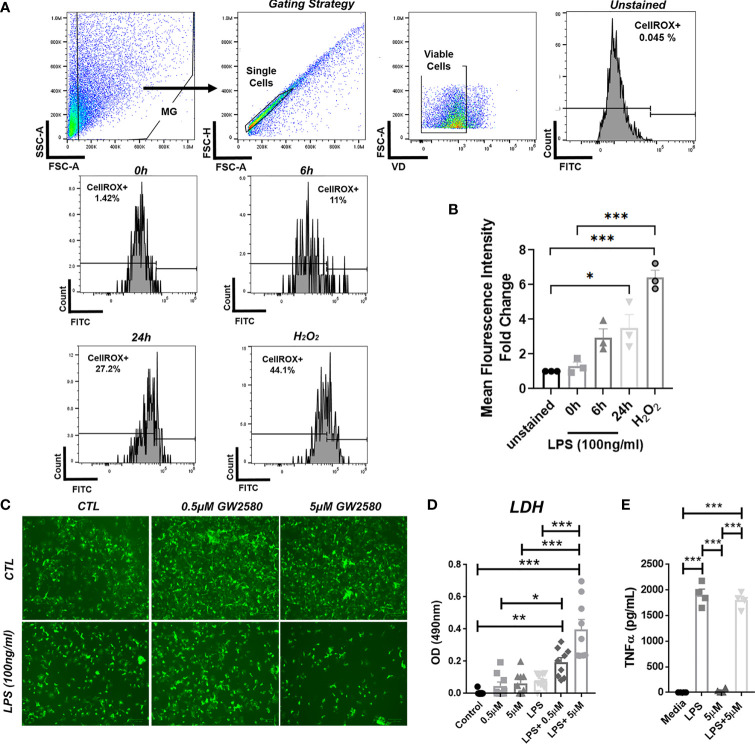
Combined treatment of primary microglia with GW2580 and LPS decreased viability *in vitro*. **(A, B)**, Primary microglia were stimulated with LPS (100ng/ml) for 0, 6 or 24h or with 75µM of H_2_O_2_, for 24h. **(A)** Microglia were stained for ROS using CellROX and then analyzed by flow cytometry to detect changes in fluorescence intensity. Gating strategy for the experiment. **(B)** Mean fluorescent intensity for ROS production after primary microglia stimulation. **(C–E)** Cells were treated with either media, LPS (100ng/ml), GW2580 (0-5µM), or LPS and GW2580 for 48h. **(C)** Representative images. **(D)** Cell death was measured by LDH assay. **(E)** TNF production by stimulated microglia. Data shown are expressed as means ± standard error (S.E.), n=3 per group for **(A)**, n=9 per group for **(B)** and n=4 per group for **(C)**. Statistical significance is represented by p-value < 0.05 (*), p-value < 0.01 (**) and p-value < 0.001 (***).

Stimulation with LPS upregulates ROS production in BV-2 cells (60) and primary microglia (61). In order to validate ROS production by primary microglia after LPS stimulation, we analyzed ROS production by flow cytometry using CellRox. For these experiments treatment with H_2_O_2_ served as a positive control. As reported by others, we found that both LPS and H_2_O_2_ treated microglia displayed increased fluorescence after 24h (**Figures 6A, B**). Since our RNA-seq data suggested that GW2580 suppressed genes associated with regulation of ROS, we questioned the effects of treatment on primary cultures stimulated with LPS. For these experiments microglia cultures were stimulated with either media, LPS, GW2580 or LPS and GW2580 together. Treatment with GW2580 or LPS alone had minimal effects on cell viability. In contrast, when microglia were concurrently treated with LPS and GW2580 we observed a marked decrease in cell viability which was validated by increased levels of LDH in the supernatant (**Figures 6C, D**). Notably, this effect was dependent on the concentration of GW2580 added to the culture (**Figure 6D**). As reported for primary peritoneal macrophages treatment did not affect the acute production of TNF from stimulated cultures (**Figure 6E**) (40). Taken together, these data indicate that LPS induces ROS in primary microglia and that CSF1R antagonism decreases viability when microglia are activated by LPS.

**Figure 7:**
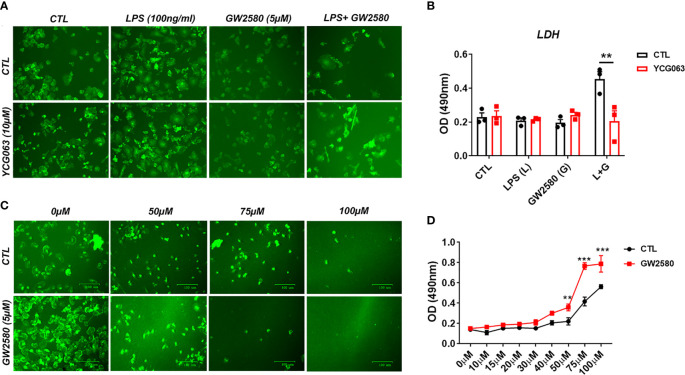
Treatment with the ROS inhibitor YCG063 inhibits cell death in GW2580 and LPS treated cultures. **(A)** Microglia treatment with YCG063, a ROS inhibitor, was used to inhibit cell death. **(B)** LDH results for both conditions. **(C)** Primary microglia were stimulated with hydrogen peroxide (from 0µM- 100µM) with and without 5µM of GW2580. **(D)** LDH results for these conditions. Four independent cultures, represented by a total 19 neonatal pups were used for **(A, B)**. **(C, D)** Three independent experiments, in at least duplicate for each condition in **(C, D)**, represented by 17 neonatal pups. Data shown are expressed as means ± standard error (SE). Statistical significance is represented by p-value < 0.01 (**) and p-value < 0.001 (***).

Next, we sought to examine a potential relationship between cell death resulting from treatment with LPS and GW2580 and ROS production. To determine if ROS was responsible for microglia death, we replicated the experimental design from previous experiments and pretreated cultures with or without YCG063, a ROS scavenger (62–64). As in previous experiments, microglial cell viability was decreased only when they were cultured in the presence of GW2580 and LPS. This effect was prevented in the presence of the ROS inhibitor YCG063 (**Figures 7A, B**), indicating that ROS was involved in the reduction of cell viability observed when microglia were treated with GW2580 and LPS.

To determine if pretreatment with GW2580 increased the sensitivity of microglia to ROS, cultures were challenged with increasing concentration of H_2_O_2_ in the presence or absence of drug treatment. Microglia pretreated with GW2580 had increased supernatant levels of LDH when stimulated with 50µM, 75µM and 100µM concentrations of H_2_O_2_ compared to control cultures (**Figures 7C, D**), indicating that GW2580 increased susceptibility of cells to a direct ROS challenge. Taken together, these data indicate that CSF1 antagonism by GW2580 suppresses the capacity for microglia to respond to oxidative stressors and sensitizes them to ROS when they become reactive.

### Treatment With the RIP1 Inhibitor Nec-1 Prevented GW2580-Mediated Toxicity

**Figure 8:**
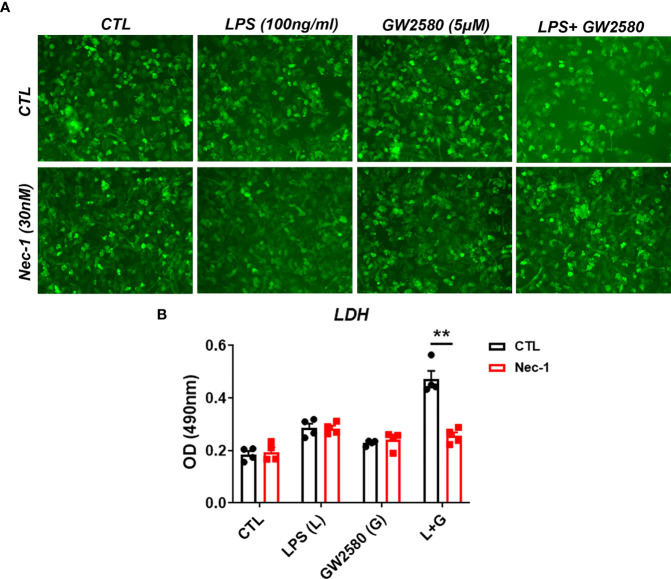
Treatment with the RIP1 inhibitor Nec-1 inhibits cell death in GW2580 and LPS treated cultures. **(A, B)** Primary microglia were treated with either media, LPS (100ng/ml), GW2580 (5µM), or LPS and GW2580 with and without Nec-1 (30nM) for 48h. **(A)** Microglia treatment with Nec-1, inhibitor of necroptosis, was used to inhibit cell death. **(B)** Cell death was addressed by LDH measurement. Data shown is expressed as means ± standard error (SE). Statistical significance is represented by p-value < 0.01 (**).

Treatment mediated dysregulation of ROS may promote cell death by either apoptosis or necroptosis (65–67). However, a time course experiment showed that treatment did not cause appreciable changes in the number of cells positively stained for cleaved caspase-3, indicating apoptosis is not likely to be the mechanism of cell death (data not shown). It was recently shown that Toll-like receptor activation in the context of caspase-8 inhibition caused microglia cell death by necroptosis in a manner contingent upon activation of RIP1/RIP3 complex and ROS production (66). CSF1R ligation by CSF1 also promotes caspase-8 activation (68), which may function to inhibit RIP1/RIP3 mediated necroptosis. To test whether necroptosis was involved in cell death resulting from co-treatment with LPS and GW2580, primary microglia were stimulated as before in the presence or absence of Nec-1, a RIP1 antagonist and potent necroptosis inhibitor (**Figure 8A**). Our data show that treatment with Nec-1 inhibited cell death in LPS and GW2580 stimulated cells (**Figure 8B**), indicating an involvement of necroptosis in treatment mediated cell death.

## Discussion

In the current study we characterized the effects of GW2580, a CSF1R antagonist, on microglia. Herein, we report that treatment does not affect weight, food disappearance or burrowing activity. However, GW2580 induced morphological changes to microglia and altered their transcriptome without affecting viability. Ontology analysis of down regulated genes indicated glutathione metabolic processes and response to oxidative stress may be dysregulated in microglia isolated from treated mice. Indeed, our culture experiments revealed that primary microglia produce ROS after stimulation with LPS, and that cell viability was only reduced under culture conditions in which cells were concurrently treated with GW2580 and LPS, an effect that was ameliorated in the presence of an ROS inhibitor. In addition, we found that GW2580 treated microglia were more sensitive to hydrogen peroxide treatment. In addition, we observed that this effect was abolished in the presence of the necroptosis inhibitor, Nec-1. These data collectively indicate that CSF1R antagonism alters the ability of microglia to regulate oxidative stress, and that concurrent treatment with LPS and GW2580 promotes cell death in a manner contingent on activation of necroptosis.

Our results indicate that eight days of treatment with GW2580 does not affect microglia cell number, burrowing behavior or food disappearance, but evokes subtle changes to microglia morphology. The effects of GW2580 on animal behavior are similar to those reported previously with PLX3397 (37, 44, 69). In fact, previous studies using the microglia depleting CSF1R antagonist PLX3397, demonstrated that treatment does not alter animal behavior in otherwise healthy animals (28, 57). In addition to this, CSF1R inhibition using PLX5622 did not alter body weights (70, 71). Furthermore PLX5622 mediated microglia depletion only altered food intake when fed a diet high in saturated fatty acids, but did not impact food intake under normal conditions (72). In terms of morphological changes, few studies have investigated the effects of systemic treatment with non-depleting CSF1R agonists on microglia morphology. Elmore et al., showed that treatment with PLX3397 depletes microglia and that microglia are only morphologically distinct from controls during the repopulation period (57). Treatment with the same antagonist caused morphological changes after 2 days of treatment (73). Similar effects were observed following PLX5622 treatment (74–76). Given that PLX3397 and PLX5622 both rapidly induce microglial cell death, the finding that these drugs alter morphology might be anticipated. Of relevance, treatment with ki20227 is similar to GW2580 in that it does not alter microglial cell numbers. However, as in our study treatment with ki20227 altered the number of microglial segments and branch endpoints (77). Finally, two additional studies have shown that GW2580 is capable of altering microglial morphology (28, 39). Taken together these data indicate that CSF1R inhibition impacts microglia morphology, and indicate that even the non-toxic molecules affect normal microglial physiology.

To better understand how GW2580 treatment affected microglial physiology, we performed an RNA-sequencing experiment on microglia isolated from the brains of control and GW2580 treated mice. Our findings revealed that treatment caused transcriptomic changes to microglia. Unsurprisingly, our data show that *Csf1* was upregulated in the GW2580 treated group, and that *Csf1r* expression exhibits a trend towards being increased. The upregulation of *Csf1* likely reflects an attempt by microglia to increase CSF1R signaling, and indicates that treatment may perturb the CSF1/CSF1R pathway. While many studies have recently utilized pharmacological inhibition of CSFR1 to delete microglia, the effects of CSF1R inhibition on the microglia transcriptome has not been completely elucidated. Similar to our results, data obtained from an RNA sequencing experiment performed on microglia isolated from wild-type and *Csf1r^-/-^* zebrafish, indicated that *Csf1r* expression was increased (78). Additionally, in this zebrafish model, upregulation of genes involved in chemotaxis and migration was observed in *Csf1r*-deficient microglia compared to wild-type controls (78). Ontology terms from our upregulated genes associated with treatment showed genes related to G-coupled receptor signaling pathway and sensory perception of smell. However, the RMPK level of the genes associated with these ontology terms was low in the control group. Oosterhof and coworkers also observed downregulation of genes related to central nervous system development and ion transportation (78), consistent with the findings from our current study. Transcriptomic analyses performed on metastatic tumors from mice treated with AZD7507, another CSF1R pharmacological inhibitor, showed alterations in tumor microenvironment composition and function (79). This does not imply any significance in terms of direct changes in the macrophage population but it suggests that macrophages present in the tumor responded to treatment and altered their relationship with the tumor, because they express CSF1R. Of relevance, human glioblastoma cells also produce CSF1 (80) and treatment with GW2580 inhibits tumor growth following implantation (81).

Transcriptomic analysis of our RNA-sequencing data showed that treatment with GW2580 decreased expression of genes involved in glutathione metabolism and regulation of ROS. We are not aware of other studies that have reported this effect of GW2580 on murine microglia. However, RNA sequencing from *Csf1r*-deficient microglia obtained from zebrafish also showed downregulation of ROS-related genes. These include *Gpx4a* (glutathione peroxidase 4a- down 516), *Mgst2* (microsomal glutathione S-transferase 2), *Gstt1a* (glutathione S-transferase theta 1a), *Gstp1* (glutathione S-transferase pi 1), *Gpx1a* (glutathione peroxidase 1a), *Nrros* (negative regulator of reactive oxygen species), and *Romo1* (reactive oxygen species modulator 1) (78) which are related to glutathione metabolism. Intriguingly, Mac2^+^ microglia that repopulate the brain after PLX5622 mediated deletion upregulate genes related to the ROS pathway (82). This finding is interesting given that intracellular ROS production appears to control the fate of common myeloid progenitors (CMP) in bone marrow. Specifically, CMP cells expressing low levels of ROS differentiate into megakaryocyte-erythrocyte progenitors and those expressing high levels of ROS upregulate CSFR1 and are likely to differentiate into granulocytes or monocytes (83). Continual stimulation of CSF1R by CSF1 inhibits receptor activator of nuclear factor-κB ligand (RANKL), activation of Nox1 and Nox4 protein expression and subsequent osteoclast generation (84). In addition to this, it has been previously proposed that CSF1 increase ROS production *via* NADPH oxidase inducing the expression of RANK (85, 86). Increased intracellular ROS activates both Akt1 and p38 MAPK pathways to regulate monocyte survival (86). Stimulation with TNF seem to upregulate CSF1 production, but in the absence of it, classical monocyte survival factors, including CSF1, GM-CSF and IL-34 seem to not contribute to cell survival (87). In addition, RNA-sequencing performed on microglia isolated from brain and spinal cords of mice treated with the CSF1R inhibitor BLZ945 show that treatment downregulated *Gstm1* (glutathione S-transferase, mu 1) and *Gsto1* (glutathione S-transferase omega 1) (88). Together these data implicate a yet to be established connection between CSF1R signaling, ROS production, detoxification, microglia differentiation and survival.

Since ROS plays an important role in microglia activation and CNS damage this provides a potential mechanism of how CSF1R inhibition, specifically GW2580, works. This becomes very relevant because it was previously found that CSF1 signaling may contribute to the pathology of neurological disorders due to the altered microglia (89). For example, a large-scale RNA-sequencing from spinal cord tissue identified CSF1R as a key node of disease progression in a model of progressive multiple sclerosis (58). They generated a selective CNS-penetrant CSF1R inhibitor molecule and observed attenuation of the inflammatory response in microglia and macrophages and significant reduction of symptoms in the animal model for multiple sclerosis (58). Similar findings have been reported with other CSF1R inhibitors to treat models of multiple sclerosis (42, 70, 90) demyelination (91–94), Alzheimer’s disease (36, 44, 91, 95, 96) neuroinflammation and neurodegeneration (36, 37, 44).

While it is established that CSF1R inhibition readily causes microglia cell death, the mechanism by which the cells die is not clearly established and may be context dependent. For instance, caspase-3^+^ microglia have been detected in naïve mice treated with the CSF1R inhibitor PLX3397 (28). Our data suggest that TLR4 activation *via* LPS stimulation in the presence of GW2580, promotes necroptosis, at least in culture. While growth factor withdrawal is known to promote apoptosis, it has also been shown that interactions between CSF1 and its receptor CSF1R leads to caspase-8 activation *via* activation of phosphatidylinosol-3 and AKT (68). Caspase 8 is an established inhibitor of the RIP1/RIP3 complex and thus reductions in caspase 8 promote cell death by necroptosis. For instance, TLR4 activation induced microglial necroptosis in a TNF/TNFR1 independent manner that was instead dependent on TRIF and RIP3 signaling (66). TLR4 activation recruits the adaptor molecules TRIF and MyD88, TRIF interacts with RIP1 and RIP3 *via* receptor-interacting protein homotypic interaction motif leading to RIPK1 ubiquitination and the formation of RIP1/RIP3 complex directing necrosome formation and necroptosis (66, 97). It is notable that LPS-activated microglia are capable of producing ROS, and that activation of the RIP1/RIP3 complex can also lead to ROS production (66). ROS can activate the necrosome causing necroptosis. As such, inhibition of ROS may prevent necroptosis (66), which relate to our findings as well. In contrast, multiple studies have shown CSF1R inhibition promotes cell death *via* apoptosis (28, 58, 75). However, our data suggest that inhibition of CSF1R signaling during a state of activation may lead to necroptosis, an effect that should be investigated further.

In summary, we have characterized the effects of systemic GW2580 treatment, a CSF1R antagonist, on microglia isolated from healthy mice. While treatment did not alter behavior or microglial cell viability, the data reveal that it suppressed expression of genes involved in regulating ROS. Culture experiments indicate that CSF1R inhibition sensitizes microglia to ROS-mediated cell death. These data indicate a yet to be established link between CSF1R signaling, redox status and microglial cell viability.

## Data Availability Statement

The data presented in the study are deposited in the Gene Expression Omnibus repository, accession number GSE185564.

## Ethics Statement

The animal study was reviewed and approved by Institutional Animal Care and Use Committee at the University of Illinois Urbana–Champaign.

## Author Contributions

KS-D, DBM, RWJ, HRG and AJS designed and coordinated the experiments. KS-D performed the experiments. MV-R performed bioinformatics statistical analysis. AYL performed the imaging and analysis. KS-D and AJS drafted the manuscript with inputs from all authors. All authors contributed to the article and approved the submitted version.

## Funding

This research was funded by grants from the National Multiple Sclerosis Society (RG 1807-32053; AJS), the NIH (RF1AG059622; RWJ), the USDA National Institute of Food and Agriculture, Hatch project ILLU-538-941 and by Division of Nutritional Sciences-Vision 20/20 (DBM).

## Conflict of Interest

The authors declare that the research was conducted in the absence of any commercial or financial relationships that could be construed as a potential conflict of interest.

## Publisher’s Note

All claims expressed in this article are solely those of the authors and do not necessarily represent those of their affiliated organizations, or those of the publisher, the editors and the reviewers. Any product that may be evaluated in this article, or claim that may be made by its manufacturer, is not guaranteed or endorsed by the publisher.

## Supplementary Material

Supplementary Figure 1 Effect of GW2580 treatment on mouse weight and behavior per gender. **(A–C)** Mice were treated with GW2580 at a dose of 80mg/kg/d by oral gavage for 8 days. The effects of treatment on weight **(A)**, food disappearance **(B)**, and burrowing activity **(C)** are shown. Data are expressed as means ± standard error (S.E.). Each group is composed of n=12-15 mice per group from four independent experiments for weight change, n=3 per group from a single experiment for food disappearance, and n=3 per group from three independent experiments for the burrowing activity.

Supplementary Figure 2 Effect of GW2580 treatment in microglia morphology. **(A–E)** Mice were treated with GW2580 by oral gavage for 8 days and the effect of treatment on microglia morphology and viability examined. **(A, B)** Representative three dimensional reconstruction of a microglia cell in the cortex of vehicle **(A)** and GW2580 treated **(B)** mice from a 40x picture. Scale bar is 10μm. **(C)** Results from Imaris analysis. Each point represents an average of three cells per mouse. Each point represents an average of three pictures. Data shown is expressed as means ± standard error (SE). *p-value < 0.05.

Supplementary Figure 3 Effect of GW2580 treatment on expression of select genes by RT-qPCR. **(A–E)**, Primary microglia were cultured from brains of neonatal mice and plated at a density of 5x10^5^ cells per well in 24-well plates. Cells were treated with GW2580 (5µM) or medium for 72h, then the expression of select genes determined by qRT-PCR. The effect of treatment on expression of *Ndufs8* **(A)**, *Prdx2* **(B)**, *Prdx5* **(C)**, and *Gpx4* **(D)** is shown. **(E)** Cell death from the treated samples was addressed by LDH measurement. Data are combined means ± standard error (S.E.) and are from 4-6 independent cultures. ### means that the gene was undetectable. Statistical significance is represented by p-value, p-value < 0.01 (**) and p-value < 0.001 (***).

## Abbreviations

AST, Astrocytes; CNS, Central nervous system; CSF1, Colony stimulating factor 1; CSFR1, Colony stimulating factor 1 receptor; CTL, Control; DMEM, *Dulbecco’s Modified Eagle Medium*; Endo, Endothelial cells; HBSS, Hanks Balanced Salt Solution; LDH, Lactate dehydrogenase; LPS, Lipopolysaccharide; MG, Microglia; Neu, Neuron; OL, Oligodendrocytes; ROS, Reactive oxygen species.

## References

[1] Paolicelli RC Jawaid A Henstridge CM Valeri A Merlini M Robinson JL. TDP-43 Depletion in Microglia Promotes Amyloid Clearance But Also Induces Synapse Loss. Neuron (2017) 95:297–308.e6. doi: 10.1016/j.neuron.2017.05.037 28669544 PMC5519492

[2] Marin-Teva JL Dusart I Colin C Gervais A van Rooijen N Mallat M. Microglia Promote the Death of Developing Purkinje Cells. Neuron (2004) 41:535–47. doi: 10.1016/S0896-6273(04)00069-8 14980203

[3] Soto-Diaz K Juda MB Blackmore S Walsh C Steelman AJ. TAK1 Inhibition in Mouse Astrocyte Cultures Ameliorates Cytokine-Induced Chemokine Production and Neutrophil Migration. J Neurochem (2020) 152:697–709. doi: 10.1111/jnc.14930 31782806

[4] von Bernhardi R Eugenin-von Bernhardi L Eugenin J. Microglial Cell Dysregulation in Brain Aging and Neurodegeneration. Front Aging Neurosci (2015) 7:124. doi: 10.3389/fnagi.2015.00124 26257642 PMC4507468

[5] Guevara CA Del Valle P Mercedes CR. Microglia and Reactive Oxygen Species Are Required for Behavioral Susceptibility to Chronic Social Defeat Stress. J Neurosci (2020) 40:1370–2. doi: 10.1523/JNEUROSCI.2175-19.2019 PMC7044733 32051290

[6] Simpson DSA Oliver PL. ROS Generation in Microglia: Understanding Oxidative Stress and Inflammation in Neurodegenerative Disease. Antioxidants (Basel) (2020) 9:743–70. doi: 10.3390/antiox9080743 PMC7463655 32823544

[7] Bogie JF Stinissen P Hendriks JJ. Macrophage Subsets and Microglia in Multiple Sclerosis. Acta Neuropathol (2014) 128:191–213. doi: 10.1007/s00401-014-1310-2 24952885

[8] Giunti D Parodi B Cordano C Uccelli A Kerlero de Rosbo N. Can We Switch Microglia's Phenotype to Foster Neuroprotection? Focus Multiple Sclerosis Immunol (2014) 141:328–39. doi: 10.1111/imm.12177 PMC3930371 24116890

[9] Heppner FL Ransohoff RM Becher B. Immune Attack: The Role of Inflammation in Alzheimer Disease. Nat Rev Neurosci (2015) 16:358–72. doi: 10.1038/nrn3880 25991443

[10] Benakis C Garcia-Bonilla L Iadecola C Anrather J. The Role of Microglia and Myeloid Immune Cells in Acute Cerebral Ischemia. Front Cell Neurosci (2014) 8:461. doi: 10.1016/B978-0-12-803058-5.00027-8 25642168 PMC4294142

[11] Machado V Zoller T Attaai A Spittau B. Microglia-Mediated Neuroinflammation and Neurotrophic Factor-Induced Protection in the MPTP Mouse Model of Parkinson's Disease-Lessons From Transgenic Mice. Int J Mol Sci (2016) 17:151–75. doi: 10.3390/ijms17020151 PMC4783885 26821015

[12] Radford RA Morsch M Rayner SL Cole NJ Pountney DL Chung RS. The Established and Emerging Roles of Astrocytes and Microglia in Amyotrophic Lateral Sclerosis and Frontotemporal Dementia. Front Cell Neurosci (2015) 9:414. doi: 10.3389/fncel.2015.00414 26578880 PMC4621294

[13] Petrelli F Pucci L Bezzi P. Astrocytes and Microglia and Their Potential Link With Autism Spectrum Disorders. Front Cell Neurosci (2016) 10:21. doi: 10.3389/fncel.2016.00021 26903806 PMC4751265

[14] Estes ML McAllister AK. Immune Mediators in the Brain and Peripheral Tissues in Autism Spectrum Disorder. Nat Rev Neurosci (2015) 16:469–86. doi: 10.1038/nrn3978 PMC5650494 26189694

[15] Menza M Dobkin RD Marin H Mark MH Gara M Bienfait K. The Role of Inflammatory Cytokines in Cognition and Other Non-Motor Symptoms of Parkinson's Disease. Psychosomatics (2010) 51:474–9. doi: 10.1371/journal.pone.0047387 PMC2987579 21051678

[16] Dantzer R. Cytokine, Sickness Behavior, and Depression. Immunol Allergy Clin North Am (2009) 29:247–64. doi: 10.1016/j.iac.2009.02.002 PMC2740752 19389580

[17] Wang WY Tan MS Yu JT Tan L. Role of Pro-Inflammatory Cytokines Released From Microglia in Alzheimer's Disease. Ann Transl Med (2015) 3:136. doi: 10.3978/j.issn.2305-5839.2015.03.49 26207229 PMC4486922

[18] Dik MG Jonker C Hack CE Smit JH Comijs HC Eikelenboom P. Serum Inflammatory Proteins and Cognitive Decline in Older Persons. Neurology (2005) 64:1371–7. doi: 10.1212/01.WNL.0000158281.08946.68 15851726

[19] Mrak RE Griffin WS. Potential Inflammatory Biomarkers in Alzheimer's Disease. J Alzheimers Dis (2005) 8:369–75. doi: 10.3233/JAD-2005-8406 16556968

[20] Erblich B Zhu L Etgen AM Dobrenis K Pollard JW. Absence of Colony Stimulation Factor-1 Receptor Results in Loss of Microglia, Disrupted Brain Development and Olfactory Deficits. PLoS One (2011) 6:e26317. doi: 10.1371/journal.pone.0026317 22046273 PMC3203114

[21] Patel S Player MR. Colony-Stimulating Factor-1 Receptor Inhibitors for the Treatment of Cancer and Inflammatory Disease. Curr Top Med Chem (2009) 9:599–610. doi: 10.2174/156802609789007327 19689368

[22] Lin H Lee E Hestir K Leo C Huang M Bosch E. Discovery of a Cytokine and Its Receptor by Functional Screening of the Extracellular Proteome. Science (2008) 320:807–11. doi: 10.1126/science.1154370 18467591

[23] Mun SH Park PSU Park-Min KH. The M-CSF Receptor in Osteoclasts and Beyond. Exp Mol Med (2020) 52:1239–54. doi: 10.1038/s12276-020-0484-z PMC8080670 32801364

[24] Zeisel A Munoz-Manchado AB Codeluppi S Lonnerberg P La Manno G Jureus A. Brain Structure. Cell Types in the Mouse Cortex and Hippocampus Revealed by Single-Cell RNA-Seq. Science (2015) 347:1138–42. doi: 10.1126/science.aaa1934 25700174

[25] Cahoy JD Emery B Kaushal A Foo LC Zamanian JL Christopherson KS. A Transcriptome Database for Astrocytes, Neurons, and Oligodendrocytes: A New Resource for Understanding Brain Development and Function. J Neurosci (2008) 28:264–78. doi: 10.1523/JNEUROSCI.4178-07.2008 PMC6671143 18171944

[26] Lemmon MA Schlessinger J. Cell Signaling by Receptor Tyrosine Kinases. Cell (2010) 141:1117–34. doi: 10.1016/j.cell.2010.06.011 PMC2914105 20602996

[27] Luo J Elwood F Britschgi M Villeda S Zhang H Ding Z. Colony-Stimulating Factor 1 Receptor (CSF1R) Signaling in Injured Neurons Facilitates Protection and Survival. J Exp Med (2013) 210:157–72. doi: 10.1084/jem.20120412 PMC3549715 23296467

[28] Elmore MR Najafi AR Koike MA Dagher NN Spangenberg EE Rice RA. Colony-Stimulating Factor 1 Receptor Signaling Is Necessary for Microglia Viability, Unmasking a Microglia Progenitor Cell in the Adult Brain. Neuron (2014) 82:380–97. doi: 10.1016/j.neuron.2014.02.040 PMC4161285 24742461

[29] Ye T Wang D Cai Z Tong L Chen Z Lu J. Antidepressive Properties of Macrophage-Colony Stimulating Factor in a Mouse Model of Depression Induced by Chronic Unpredictable Stress. Neuropharmacology (2020) 172:108132. doi: 10.1016/j.neuropharm.2020.108132 32407925

[30] Hagemeyer N Hanft KM Akriditou MA Unger N Park ES Stanley ER. Microglia Contribute to Normal Myelinogenesis and to Oligodendrocyte Progenitor Maintenance During Adulthood. Acta Neuropathol (2017) 134:441–58. doi: 10.1007/s00401-017-1747-1 PMC5951721 28685323

[31] Han J Harris RA Zhang XM. An Updated Assessment of Microglia Depletion: Current Concepts and Future Directions. Mol Brain (2017) 10:25. doi: 10.1186/s13041-017-0307-x 28629387 PMC5477141

[32] Yan D Kowal J Akkari L Schuhmacher AJ Huse JT West BL. Inhibition of Colony Stimulating Factor-1 Receptor Abrogates Microenvironment-Mediated Therapeutic Resistance in Gliomas. Oncogene (2017) 36:6049–58. doi: 10.1038/onc.2017.261 PMC5666319 28759044

[33] Mok S Koya RC Tsui C Xu J Robert L Wu L. Inhibition of CSF-1 Receptor Improves the Antitumor Efficacy of Adoptive Cell Transfer Immunotherapy. Cancer Res (2014) 74:153–61. doi: 10.1158/0008-5472.CAN-13-1816 PMC3947337 24247719

[34] Pyonteck SM Akkari L Schuhmacher AJ Bowman RL Sevenich L Quail DF. CSF-1R Inhibition Alters Macrophage Polarization and Blocks Glioma Progression. Nat Med (2013) 19:1264–72. doi: 10.1038/nm.3337 PMC3840724 24056773

[35] Ikegashira K Ikenogami T Yamasaki T Oka T Hase Y Miyagawa N. Optimization of an Azetidine Series as Inhibitors of Colony Stimulating Factor-1 Receptor (CSF-1r) Type II to Lead to the Clinical Candidate JTE-952. Bioorg Med Chem Lett (2019) 29:873–7. doi: 10.1016/j.bmcl.2019.02.006 30755337

[36] Mancuso R Fryatt G Cleal M Obst J Pipi E Monzon-Sandoval J. CSF1R Inhibitor JNJ-40346527 Attenuates Microglial Proliferation and Neurodegeneration in P301S Mice. Brain (2019) 142:3243–64. doi: 10.1093/brain/awz241 PMC6794948 31504240

[37] Neal ML Fleming SM Budge KM Boyle AM Kim C Alam G. Pharmacological Inhibition of CSF1R by GW2580 Reduces Microglial Proliferation and Is Protective Against Neuroinflammation and Dopaminergic Neurodegeneration. FASEB J (2020) 34:1679–94. doi: 10.1096/fj.201900567RR PMC7212500 31914683

[38] Edwards VD Sweeney DT Ho H Eide CA Rofelty A Agarwal A. Targeting of Colony-Stimulating Factor 1 Receptor (CSF1R) in the CLL Microenvironment Yields Antineoplastic Activity in Primary Patient Samples. Oncotarget (2018) 9:24576–89. doi: 10.18632/oncotarget.25191 PMC5973855 29872489

[39] Gerber YN Saint-Martin GP Bringuier CM Bartolami S Goze-Bac C Noristani HN. CSF1R Inhibition Reduces Microglia Proliferation, Promotes Tissue Preservation and Improves Motor Recovery After Spinal Cord Injury. Front Cell Neurosci (2018) 12:368. doi: 10.3389/fncel.2018.00368 30386212 PMC6198221

[40] Conway JG McDonald B Parham J Keith B Rusnak DW Shaw E. Inhibition of Colony-Stimulating-Factor-1 Signaling *In Vivo* With the Orally Bioavailable cFMS Kinase Inhibitor GW2580. Proc Natl Acad Sci USA (2005) 102:16078–83. doi: 10.1073/pnas.0502000102 PMC1276040 16249345

[41] Moughon DL He H Schokrpur S Jiang ZK Yaqoob M David J. Macrophage Blockade Using CSF1R Inhibitors Reverses the Vascular Leakage Underlying Malignant Ascites in Late-Stage Epithelial Ovarian Cancer. Cancer Res (2015) 75:4742–52. doi: 10.1158/0008-5472.CAN-14-3373 PMC4675660 26471360

[42] Borjini N Fernandez M Giardino L Calza L. Cytokine and Chemokine Alterations in Tissue, CSF, and Plasma in Early Presymptomatic Phase of Experimental Allergic Encephalomyelitis (EAE), in a Rat Model of Multiple Sclerosis. J Neuroinflamm (2016) 13:291. doi: 10.1186/s12974-016-0757-6 PMC5111339 27846891

[43] Crespo O Kang SC Daneman R Lindstrom TM Ho PP Sobel RA. Tyrosine Kinase Inhibitors Ameliorate Autoimmune Encephalomyelitis in a Mouse Model of Multiple Sclerosis. J Clin Immunol (2011) 31:1010–20. doi: 10.1007/s10875-011-9579-6 PMC3225802 21847523

[44] Olmos-Alonso A Schetters ST Sri S Askew K Mancuso R Vargas-Caballero M. Pharmacological Targeting of CSF1R Inhibits Microglial Proliferation and Prevents the Progression of Alzheimer's-Like Pathology. Brain (2016) 139:891–907. doi: 10.1093/brain/awv379 26747862 PMC4766375

[45] Martinez-Muriana A Mancuso R Francos-Quijorna I Olmos-Alonso A Osta R Perry VH. CSF1R Blockade Slows the Progression of Amyotrophic Lateral Sclerosis by Reducing Microgliosis and Invasion of Macrophages Into Peripheral Nerves. Sci Rep (2016) 6:25663. doi: 10.1038/srep25663 27174644 PMC4865981

[46] Gomez-Nicola D Fransen NL Suzzi S Perry VH. Regulation of Microglial Proliferation During Chronic Neurodegeneration. J Neurosci (2013) 33:2481–93. doi: 10.1523/JNEUROSCI.4440-12.2013 PMC6619184 23392676

[47] Chalmers SA Wen J Shum J Doerner J Herlitz L Putterman C. CSF-1R Inhibition Attenuates Renal and Neuropsychiatric Disease in Murine Lupus. Clin Immunol (2017) 185:100–8. doi: 10.1016/j.clim.2016.08.019 PMC5326697 27570219

[48] Jirkof P Cesarovic N Rettich A Nicholls F Seifert B Arras M. Burrowing Behavior as an Indicator of Post-Laparotomy Pain in Mice. Front Behav Neurosci (2010) 4:165. doi: 10.3389/fnbeh.2010.00165 21031028 PMC2965018

[49] Bolger AM Lohse M Usadel B. Trimmomatic: A Flexible Trimmer for Illumina Sequence Data. Bioinformatics (2014) 30:2114–20. doi: 10.1093/bioinformatics/btu170 PMC4103590 24695404

[50] Dobin A Davis CA Schlesinger F Drenkow J Zaleski C Jha S. STAR: Ultrafast Universal RNA-Seq Aligner. Bioinformatics (2013) 29:15–21. doi: 10.1093/bioinformatics/bts635 23104886 PMC3530905

[51] Liao Y Smyth GK Shi W. Featurecounts: An Efficient General Purpose Program for Assigning Sequence Reads to Genomic Features. Bioinformatics (2014) 30:923–30. doi: 10.1093/bioinformatics/btt656 24227677

[52] Robinson MD Oshlack A. A Scaling Normalization Method for Differential Expression Analysis of RNA-Seq Data. Genome Biol (2010) 11:R25. doi: 10.1186/gb-2010-11-3-r25 20196867 PMC2864565

[53] Steelman AJ Zhou Y Koito H Kim S Payne HR Lu QR. Activation of Oligodendroglial Stat3 Is Required for Efficient Remyelination. Neurobiol Dis (2016) 91:336–46. doi: 10.1016/j.nbd.2016.03.023 PMC4962532 27060559

[54] Shi L Jones WD Jensen RV Harris SC Perkins RG Goodsaid FM. The Balance of Reproducibility, Sensitivity, and Specificity of Lists of Differentially Expressed Genes in Microarray Studies. BMC Bioinf (2008) 9 Suppl 9:S10. doi: 10.1186/1471-2105-9-S9-S10 PMC2537561 18793455

[55] Guo L Lobenhofer EK Wang C Shippy R Harris SC Zhang L. Rat Toxicogenomic Study Reveals Analytical Consistency Across Microarray Platforms. Nat Biotechnol (2006) 24:1162–9. doi: 10.1038/nbt1238 17061323

[56] Tabula Muris C. C. Overall, C. Logistical, C. Organ, Processing, P. Library, Sequencing, a. Computational Data, a. Cell Type, G. Writing, G. Supplemental Text Writing, and I. Principal, Single-Cell Transcriptomics of 20 Mouse Organs Creates a Tabula Muris. Nature (2018) 562:367–72. doi: 10.1038/s41586-018-0590-4 PMC6642641 30283141

[57] Elmore MR Lee RJ West BL Green KN. Characterizing Newly Repopulated Microglia in the Adult Mouse: Impacts on Animal Behavior, Cell Morphology, and Neuroinflammation. PLoS One (2015) 10:e0122912. doi: 10.1371/journal.pone.0122912 25849463 PMC4388515

[58] Hagan N Kane JL Grover D Woodworth L Madore C Saleh J. CSF1R Signaling Is a Regulator of Pathogenesis in Progressive MS. Cell Death Dis (2020) 11:904. doi: 10.1038/s41419-020-03084-7 33097690 PMC7584629

[59] Smith AM Gibbons HM Oldfield RL Bergin PM Mee EW Curtis MA. M-CSF Increases Proliferation and Phagocytosis While Modulating Receptor and Transcription Factor Expression in Adult Human Microglia. J Neuroinflamm (2013) 10:85. doi: 10.1186/1742-2094-10-85 PMC3729740 23866312

[60] Sun GY Li R Cui J Hannink M Gu Z Fritsche KL. Withania Somnifera and Its Withanolides Attenuate Oxidative and Inflammatory Responses and Up-Regulate Antioxidant Responses in BV-2 Microglial Cells. Neuromolecular Med (2016) 18:241–52. doi: 10.1007/s12017-016-8411-0 27209361

[61] Yauger YJ Bermudez S Moritz KE Glaser E Stoica B Byrnes KR. Iron Accentuated Reactive Oxygen Species Release by NADPH Oxidase in Activated Microglia Contributes to Oxidative Stress In Vitro. J Neuroinflamm (2019) 16:41. doi: 10.1186/s12974-019-1430-7 PMC6378754 30777083

[62] Paeng SH Park WS Jung WK Lee DS Kim GY Choi YH. YCG063 Inhibits Pseudomonas Aeruginosa LPS-Induced Inflammation in Human Retinal Pigment Epithelial Cells Through the TLR2-Mediated AKT/NF-kappaB Pathway and ROS-Independent Pathways. Int J Mol Med (2015) 36:808–16. doi: 10.3892/ijmm.2015.2266 26136104

[63] Locatelli SL Cleris L Stirparo GG Tartari S Saba E Pierdominici M. BIM Upregulation and ROS-Dependent Necroptosis Mediate the Antitumor Effects of the HDACi Givinostat and Sorafenib in Hodgkin Lymphoma Cell Line Xenografts. Leukemia (2014) 28:1861–71. doi: 10.1038/leu.2014.81 24561519

[64] Kim KH Park JY Jung HJ Kwon HJ. Identification and Biological Activities of a New Antiangiogenic Small Molecule That Suppresses Mitochondrial Reactive Oxygen Species. Biochem Biophys Res Commun (2011) 404:541–5. doi: 10.1016/j.bbrc.2010.12.022 21144820

[65] Redza-Dutordoir M Averill-Bates DA. Activation of Apoptosis Signalling Pathways by Reactive Oxygen Species. Biochim Biophys Acta (2016) 1863:2977–92. doi: 10.1016/j.bbamcr.2016.09.012 27646922

[66] Kim SJ Li J. Caspase Blockade Induces RIP3-Mediated Programmed Necrosis in Toll-Like Receptor-Activated Microglia. Cell Death Dis (2013) 4:e716. doi: 10.1038/cddis.2013.238 23846218 PMC3730412

[67] Ryter SW Kim HP Hoetzel A Park JW Nakahira K Wang X. Mechanisms of Cell Death in Oxidative Stress. Antioxid Redox Signal (2007) 9:49–89. doi: 10.1089/ars.2007.9.49 17115887

[68] Jacquel A Benikhlef N Paggetti J Lalaoui N Guery L Dufour EK. Colony-Stimulating Factor-1-Induced Oscillations in Phosphatidylinositol-3 Kinase/AKT Are Required for Caspase Activation in Monocytes Undergoing Differentiation Into Macrophages. Blood (2009) 114:3633–41. doi: 10.1182/blood-2009-03-208843 19721010

[69] Shaposhnik Z Wang X Lusis AJ. Arterial Colony Stimulating Factor-1 Influences Atherosclerotic Lesions by Regulating Monocyte Migration and Apoptosis. J Lipid Res (2010) 51:1962–70. doi: 10.1194/jlr.M005215 PMC2882747 20194110

[70] Nissen JC Thompson KK West BL Tsirka SE. Csf1R Inhibition Attenuates Experimental Autoimmune Encephalomyelitis and Promotes Recovery. Exp Neurol (2018) 307:24–36. doi: 10.1016/j.expneurol.2018.05.021 29803827 PMC6380683

[71] Seitz S Clarke P Tyler KL. Pharmacologic Depletion of Microglia Increases Viral Load in the Brain and Enhances Mortality in Murine Models of Flavivirus-Induced Encephalitis. J Virol (2018) 92:JVI.00525–18–48. doi: 10.1128/JVI.00525-18 PMC6069207 29899084

[72] Valdearcos M Robblee MM Benjamin DI Nomura DK Xu AW Koliwad SK. Microglia Dictate the Impact of Saturated Fat Consumption on Hypothalamic Inflammation and Neuronal Function. Cell Rep (2014) 9:2124–38. doi: 10.1016/j.celrep.2014.11.018 PMC4617309 25497089

[73] De I Nikodemova M Steffen MD Sokn E Maklakova VI Watters JJ. CSF1 Overexpression has Pleiotropic Effects on Microglia In Vivo. Glia (2014) 62:1955–67. doi: 10.1002/glia.22717 PMC4205273 25042473

[74] Ali S Mansour AG Huang W Queen NJ Mo X Anderson JM. CSF1R Inhibitor PLX5622 and Environmental Enrichment Additively Improve Metabolic Outcomes in Middle-Aged Female Mice. Aging (Albany NY) (2020) 12:2101–22. doi: 10.18632/aging.102724 PMC7041757 32007953

[75] Henry RJ Ritzel RM Barrett JP Doran SJ Jiao Y Leach JB. Microglial Depletion With CSF1R Inhibitor During Chronic Phase of Experimental Traumatic Brain Injury Reduces Neurodegeneration and Neurological Deficits. J Neurosci (2020) 40:2960–74. doi: 10.1523/JNEUROSCI.2402-19.2020 PMC7117897 32094203

[76] Yang X Zhao L Campos MM Abu-Asab M Ortolan D Hotaling N. CSF1R Blockade Induces Macrophage Ablation and Results in Mouse Choroidal Vascular Atrophy and RPE Disorganization. Elife (2020) 9:1–30. doi: 10.7554/eLife.55564 PMC7156269 32234210

[77] Hou B Jiang C Wang D Wang G Wang Z Zhu M. Pharmacological Targeting of CSF1R Inhibits Microglial Proliferation and Aggravates the Progression of Cerebral Ischemic Pathology. Front Cell Neurosci (2020) 14:267. doi: 10.3389/fncel.2020.00267 33177990 PMC7596178

[78] Oosterhof N Kuil LE van der Linde HC Burm SM Berdowski W van Ijcken WFJ. Colony-Stimulating Factor 1 Receptor (CSF1R) Regulates Microglia Density and Distribution, But Not Microglia Differentiation In Vivo. Cell Rep (2018) 24:1203–17.e6. doi: 10.1016/j.celrep.2018.06.113 30067976

[79] Candido JB Morton JP Bailey P Campbell AD Karim SA Jamieson T. CSF1R(+) Macrophages Sustain Pancreatic Tumor Growth Through T Cell Suppression and Maintenance of Key Gene Programs That Define the Squamous Subtype. Cell Rep (2018) 23:1448–60. doi: 10.1016/j.celrep.2018.03.131 PMC5946718 29719257

[80] De I Steffen MD Clark PA Patros CJ Sokn E Bishop SM. CSF1 Overexpression Promotes High-Grade Glioma Formation Without Impacting the Polarization Status of Glioma-Associated Microglia and Macrophages. Cancer Res (2016) 76:2552–60. doi: 10.1158/0008-5472.CAN-15-2386 PMC4873447 27013192

[81] Achyut BR Shankar A Iskander AS Ara R Angara K Zeng P. Bone Marrow Derived Myeloid Cells Orchestrate Antiangiogenic Resistance in Glioblastoma Through Coordinated Molecular Networks. Cancer Lett (2015) 369:416–26. doi: 10.1016/j.canlet.2015.09.004 PMC4686232 26404753

[82] Zhan L Fan L Kodama L Sohn PD Wong MY Mousa GA. A MAC2-Positive Progenitor-Like Microglial Population is Resistant to CSF1R Inhibition in Adult Mouse Brain. Elife (2020) 9:1–22. doi: 10.7554/eLife.51796 PMC7591254 33054973

[83] Shinohara A Imai Y Nakagawa M Takahashi T Ichikawa M Kurokawa M. Intracellular Reactive Oxygen Species Mark and Influence the Megakaryocyte-Erythrocyte Progenitor Fate of Common Myeloid Progenitors. Stem Cells (2014) 32:548–57. doi: 10.1002/stem.1588 24167091

[84] Wittrant Y Gorin Y Mohan S Wagner B Abboud-Werner SL. Colony-Stimulating Factor-1 (CSF-1) Directly Inhibits Receptor Activator of Nuclear Factor-{Kappa}B Ligand (RANKL) Expression by Osteoblasts. Endocrinology (2009) 150:4977–88. doi: 10.1210/en.2009-0248 PMC2775986 19819976

[85] Nakanishi A Hie M Iitsuka N Tsukamoto I. A Crucial Role for Reactive Oxygen Species in Macrophage Colony-Stimulating Factor-Induced RANK Expression in Osteoclastic Differentiation. Int J Mol Med (2013) 31:874–80. doi: 10.3892/ijmm.2013.1258 23443487

[86] Wang Y Zeigler MM Lam GK Hunter MG Eubank TD Khramtsov VV. The Role of the NADPH Oxidase Complex, P38 MAPK, and Akt in Regulating Human Monocyte/Macrophage Survival. Am J Respir Cell Mol Biol (2007) 36:68–77. doi: 10.1165/rcmb.2006-0165OC 16931806 PMC1899309

[87] Darrieutort-Laffite C Boutet MA Chatelais M Brion R Blanchard F Heymann D. IL-1beta and TNFalpha Promote Monocyte Viability Through the Induction of GM-CSF Expression by Rheumatoid Arthritis Synovial Fibroblasts. Mediators Inflamm (2014) 2014:241840. doi: 10.1155/2014/241840 25484525 PMC4251793

[88] Giorgetti E Panesar M Zhang Y Joller S Ronco M Obrecht M. Modulation of Microglia by Voluntary Exercise or CSF1R Inhibition Prevents Age-Related Loss of Functional Motor Units. Cell Rep (2019) 29:1539–54.e7. doi: 10.1016/j.celrep.2019.10.003 31693894

[89] Chitu V Biundo F Shlager GGL Park ES Wang P Gulinello ME. Microglial Homeostasis Requires Balanced CSF-1/CSF-2 Receptor Signaling. Cell Rep (2020) 30:3004–19.e5. doi: 10.1016/j.celrep.2020.02.028 32130903 PMC7370656

[90] Wlodarczyk A Benmamar-Badel A Cedile O Jensen KN Kramer I Elsborg NB. CSF1R Stimulation Promotes Increased Neuroprotection by CD11c+ Microglia in EAE. Front Cell Neurosci (2018) 12:523. doi: 10.3389/fncel.2018.00523 30687013 PMC6335250

[91] Pons V Levesque P Plante MM Rivest S. Conditional Genetic Deletion of CSF1 Receptor in Microglia Ameliorates the Physiopathology of Alzheimer's Disease. Alzheimers Res Ther (2021) 13:8. doi: 10.1186/s13195-020-00747-7 33402196 PMC7783991

[92] Liu Y Given KS Dickson EL Owens GP Macklin WB Bennett JL. Concentration-Dependent Effects of CSF1R Inhibitors on Oligodendrocyte Progenitor Cells Ex Vivo and In Vivo. Exp Neurol (2019) 318:32–41. doi: 10.1016/j.expneurol.2019.04.011 31029597 PMC6615458

[93] Tahmasebi F Pasbakhsh P Mortezaee K Madadi S Barati S Kashani IR. Effect of the CSF1R Inhibitor PLX3397 on Remyelination of Corpus Callosum in a Cuprizone-Induced Demyelination Mouse Model. J Cell Biochem (2019) 120:10576–86. doi: 10.1002/jcb.28344 30628737

[94] Beckmann N Giorgetti E Neuhaus A Zurbruegg S Accart N Smith P. Brain Region-Specific Enhancement of Remyelination and Prevention of Demyelination by the CSF1R Kinase Inhibitor BLZ945. Acta Neuropathol Commun (2018) 6:9. doi: 10.1186/s40478-018-0510-8 29448957 PMC5815182

[95] Martin-Estebane M Gomez-Nicola D. Targeting Microglial Population Dynamics in Alzheimer's Disease: Are We Ready for a Potential Impact on Immune Function? Front Cell Neurosci (2020) 14:149. doi: 10.3389/fncel.2020.00149 32581720 PMC7289918

[96] Sosna J Philipp S Albay R 3rd Reyes-Ruiz JM Baglietto-Vargas D LaFerla FM. Early Long-Term Administration of the CSF1R Inhibitor PLX3397 Ablates Microglia and Reduces Accumulation of Intraneuronal Amyloid, Neuritic Plaque Deposition and Pre-Fibrillar Oligomers in 5XFAD Mouse Model of Alzheimer's Disease. Mol Neurodegener (2018) 13:11. doi: 10.1186/s13024-018-0244-x 29490706 PMC5831225

[97] Seo J Nam YW Kim S Oh DB Song J. Necroptosis Molecular Mechanisms: Recent Findings Regarding Novel Necroptosis Regulators. Exp Mol Med (2021) 53:1007–17. doi: 10.1038/s12276-021-00634-7 PMC8166896 34075202

