# Supplementary figures and images for: Treatment With the CSF1R Antagonist GW2580, Sensitizes Microglia to Reactive Oxygen Species
Source: Front Immunol. 2021 Nov 26;12:734349. doi: 10.3389/fimmu.2021.734349 (PMC8664563; doi:10.3389/fimmu.2021.734349)

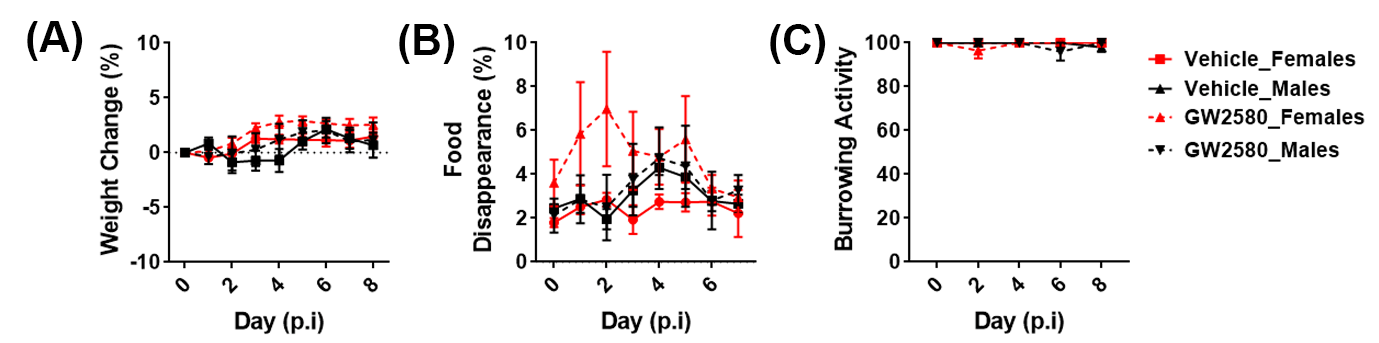

Supplement: Supplementary Figure 1 — Effect of GW2580 treatment on mouse weight and behavior per gender. (A–C) Mice were treated with GW2580 at a dose of 80mg/kg/d by oral gavage for 8 days. The effects of treatment on weight (A), food disappearance (B), and burrowing activity (C) are shown. Data are expressed as means ± standard error (S.E.). Each group is composed of n=12-15 mice per group from four independent experiments for weight change, n=3 per group from a single experiment for food disappearance, and n=3 per group from three independent experiments for the burrowing activity. [file Image_1.tif]

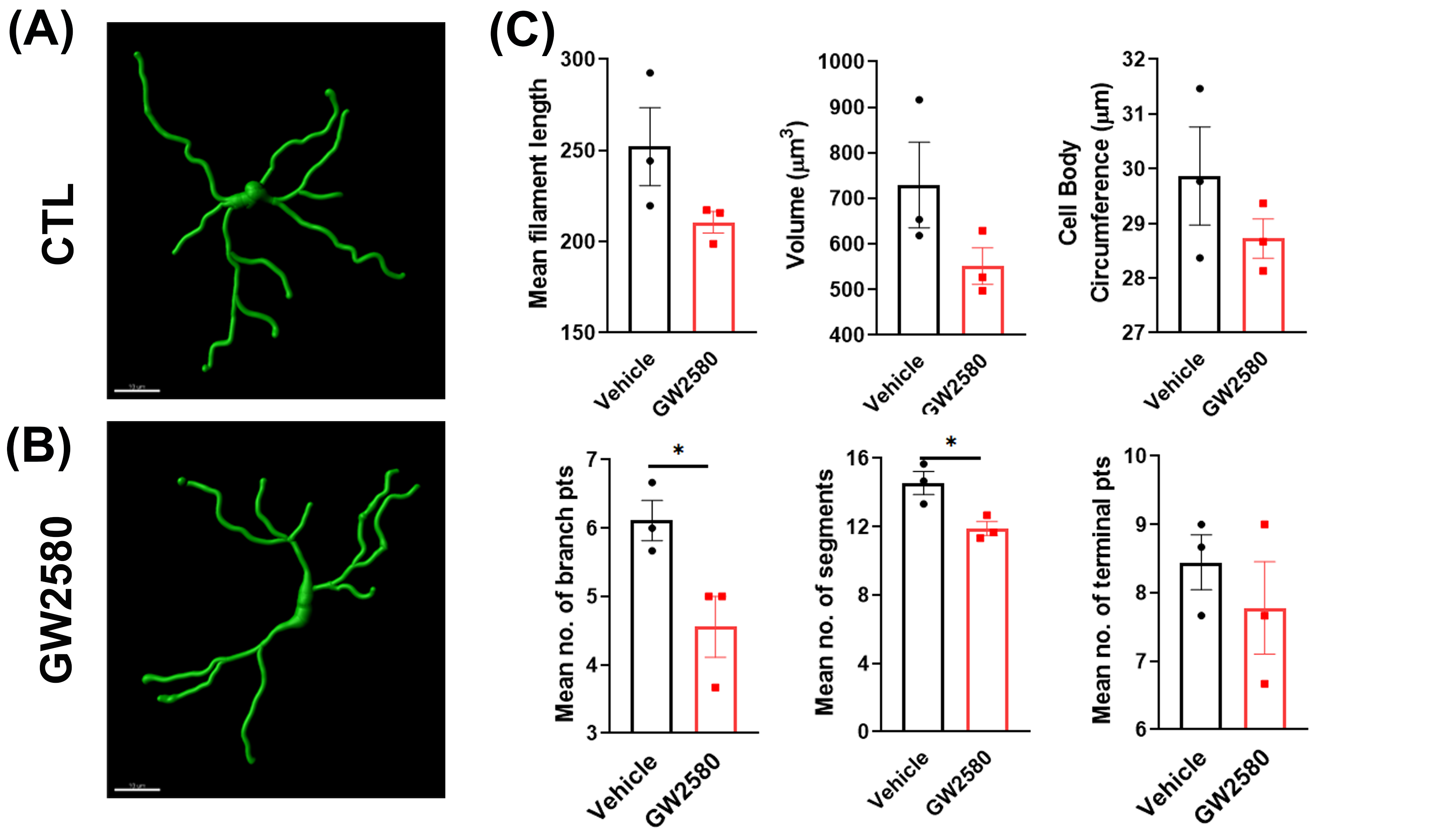

Supplement: Supplementary Figure 2 — Effect of GW2580 treatment in microglia morphology. (A–E) Mice were treated with GW2580 by oral gavage for 8 days and the effect of treatment on microglia morphology and viability examined. (A, B) Representative three dimensional reconstruction of a microglia cell in the cortex of vehicle (A) and GW2580 treated (B) mice from a 40x picture. Scale bar is 10μm. (C) Results from Imaris analysis. Each point represents an average of three cells per mouse. Each point represents an average of three pictures. Data shown is expressed as means ± standard error (SE). *p-value < 0.05. [file Image_2.tif]

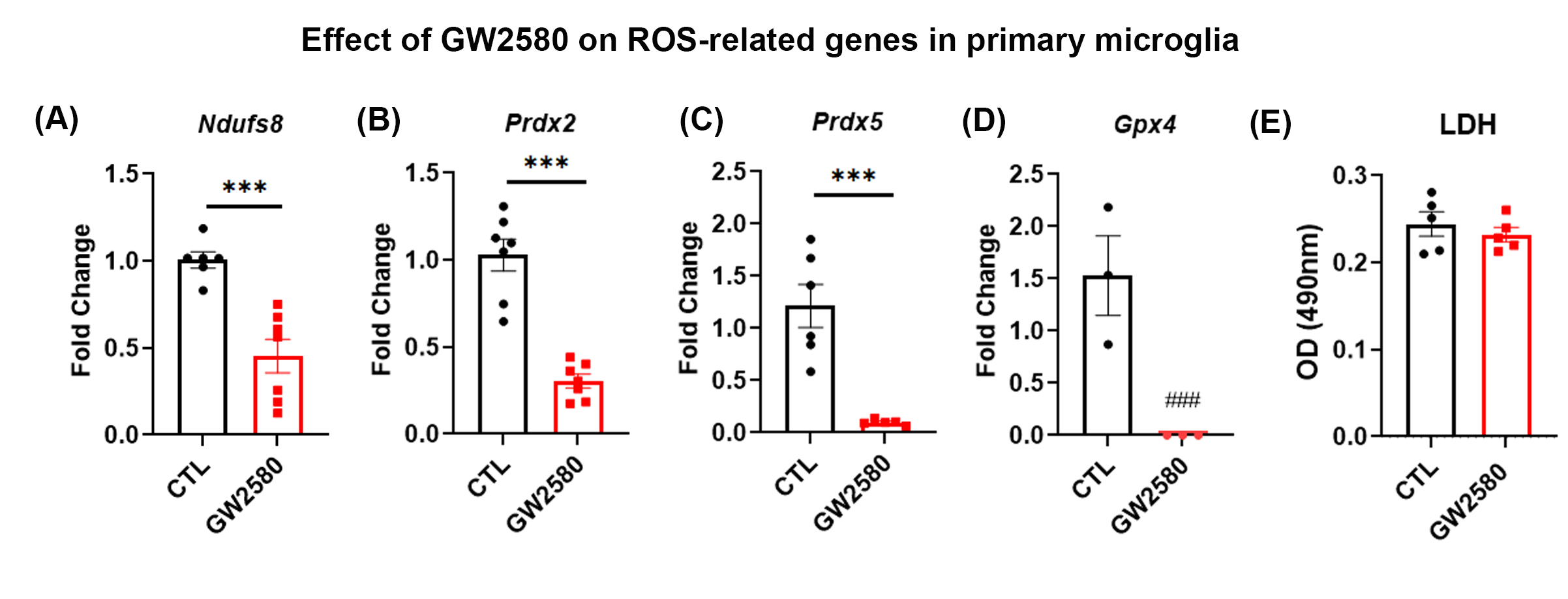

Supplement: Supplementary Figure 3 — Effect of GW2580 treatment on expression of select genes by RT-qPCR. (A–E), Primary microglia were cultured from brains of neonatal mice and plated at a density of 5x10^5^ cells per well in 24-well plates. Cells were treated with GW2580 (5µM) or medium for 72h, then the expression of select genes determined by qRT-PCR. The effect of treatment on expression of Ndufs8 (A), Prdx2 (B), Prdx5 (C), and Gpx4 (D) is shown. (E) Cell death from the treated samples was addressed by LDH measurement. Data are combined means ± standard error (S.E.) and are from 4-6 independent cultures. ### means that the gene was undetectable. Statistical significance is represented by p-value, p-value < 0.01 (**) and p-value < 0.001 (***). [file Image_3.tif]
